# *HOTAIR* interacts with PRC2 complex regulating the regional preadipocyte transcriptome and human fat distribution

**DOI:** 10.1016/j.celrep.2022.111136

**Published:** 2022-07-28

**Authors:** Feng-Chih Kuo, Matt J. Neville, Rugivan Sabaratnam, Agata Wesolowska-Andersen, Daniel Phillips, Laura B.L. Wittemans, Andrea D. van Dam, Nellie Y. Loh, Marijana Todorčević, Nathan Denton, Katherine A. Kentistou, Peter K. Joshi, Constantinos Christodoulides, Claudia Langenberg, Philippe Collas, Fredrik Karpe, Katherine E. Pinnick

**Affiliations:** 1 Oxford Centre for Diabetes, Endocrinology, and Metabolism, Radcliffe Department of Medicine, University of Oxford, Churchill Hospital, Headington OX3 7LE, UK; 2 Division of Endocrinology and Metabolism, Department of Internal Medicine, Tri-Service General Hospital, National Defence Medical Centre, Taipei, Taiwan; 3 NIHR Oxford Biomedical Research Centre, OUH Foundation Trust, Oxford, UK; 4 Institute of Clinical Research, University of Southern Denmark, 5000 Odense C, Denmark; 5 Steno Diabetes Center Odense, Odense University Hospital, 5000 Odense C, Denmark; 6 Wellcome Trust Centre for Human Genetics, University of Oxford, Roosevelt Drive, Oxford OX3 7BN, UK; 7 MRC Epidemiology Unit, Institute of Metabolic Science, University of Cambridge School of Clinical Medicine, Cambridge CB2 0QQ, UK; 8 The Big Data Institute, Li Ka Shing Centre for Health Information and Discovery, University of Oxford, Oxford, UK; 9 Centre for Global Health Research, Usher Institute, University of Edinburgh, Teviot Place, Edinburgh EH8 9AG, UK; 10 Centre for Cardiovascular Sciences, Queen’s Medical Research Institute, University of Edinburgh, Edinburgh EH16 4TJ, UK; 11 Department of Molecular Medicine, Institute of Basic Medical Sciences, Faculty of Medicine, University of Oslo, Oslo, Norway; 12 Department of Immunology, Oslo University Hospital, Oslo, Norway

**Keywords:** subcutaneous adipose tissue, fat distribution, lncRNA, *HOTAIR*, adipogenesis, epigenetic regulation

## Abstract

Mechanisms governing regional human adipose tissue (AT) development remain undefined. Here, we show that the long non-coding RNA *HOTAIR* (*HOX* transcript antisense RNA) is exclusively expressed in gluteofemoral AT, where it is essential for adipocyte development. We find that *HOTAIR* interacts with polycomb repressive complex 2 (PRC2) and we identify core *HOTAIR*-PRC2 target genes involved in adipocyte lineage determination. Repression of target genes coincides with PRC2 promoter occupancy and H3K27 trimethylation. *HOTAIR* is also involved in modifying the gluteal adipocyte transcriptome through alternative splicing. Gluteal-specific expression of *HOTAIR* is maintained by defined regions of open chromatin across the *HOTAIR* promoter. *HOTAIR* expression levels can be modified by hormonal (estrogen, glucocorticoids) and genetic variation (rs1443512 is a *HOTAIR* eQTL associated with reduced gynoid fat mass). These data identify *HOTAIR* as a dynamic regulator of the gluteal adipocyte transcriptome and epigenome with functional importance for human regional AT development.

## Highlights

• *HOTAIR* is expressed specifically in the gluteal adipose tissue depot
• *In vitro* impairment of *HOTAIR* prevents gluteal adipocyte development
• Minor allele carriers of a *HOTAIR*-lowering eQTL have reduced lower-body fat mass

## Graphical abstract

**Figure:**
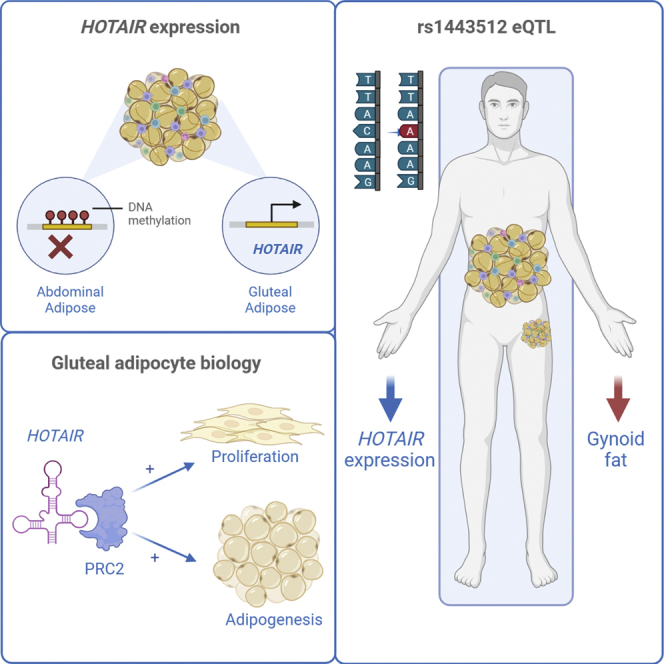

## Teaser

Kuo et al. identify *HOTAIR* as a lower-body-specific regulator of human fat tissue development. *HOTAIR* deficiency results in impaired gluteal preadipocyte proliferation and differentiation. Genetic associations in a population of 25,200 healthy individuals confirm that carriers of a *HOTAIR*-lowering genetic variant have reduced lower-body fat mass.

## Introduction

Waist-to-hip ratio (WHR), as a surrogate measure of human fat distribution, is more strongly associated with obesity-related metabolic disorders than body fatness per se (Vazquez et al., 2007; Yusuf et al., 2005). Anatomically, more than 90% of human body fat can be classified into three main depots: abdominal subcutaneous adipose tissue (ASAT), gluteofemoral subcutaneous adipose tissue (GSAT), and visceral fat. In terms of biological characteristics, GSAT possesses the ability for long-term fatty acid storage (Arner et al., 1990; Wahrenberg et al., 1989) and, in adults, responds to weight gain mainly by adipocyte hyperplasia (Tchoukalova et al., 2010), whereas ASAT stores fatty acids on a shorter-term basis and expands by adipocyte hypertrophy in response to demands for fat storage. Therefore, GSAT can be considered a metabolic sink that sequesters excess energy and prevents the deposition of ectopic fat in insulin-sensitive tissues, such as liver, muscle, and pancreas (Karpe and Pinnick, 2015). Using dual-energy X-ray absorptiometry (DXA) to precisely quantify regional fat mass, gynoid fat mass is inversely associated with cardiovascular risk factors, such as insulin resistance, dyslipidaemia, and hypertension in both genders, after adjustment for total fat mass (Pinnick et al., 2014; Wiklund et al., 2008). In this regard, determinants that regulate the functional mass of regional adipose tissue (AT), particularly the size of the gluteal depot, may offer insights into therapeutic targets for obesity-associated metabolic comorbidities.

To date, direct comparisons of transcriptome profiling between GSAT and ASAT have been performed by four independent research groups (Gehrke et al., 2013; Karastergiou et al., 2013; Passaro et al., 2017; Pinnick et al., 2014). Consistent between these studies is the finding that developmental genes are differentially expressed between the two fat depots, which includes multiple members of the homeobox (*HOX*) gene family. However, one of the most differentially expressed transcripts is a long non-coding RNA (lncRNA) named *HOX* transcript antisense RNA (*HOTAIR*), which resides within the *HOXC* cluster on chromosome 12 (Divoux et al., 2014; Pinnick et al., 2014). lncRNAs are non-protein coding transcripts more than 200 nt in length (Ma et al., 2013) that display a high-order RNA structure and are usually expressed at low levels, but with high tissue specificity (Cabili et al., 2011). lncRNAs regulate gene expression through diverse mechanisms, including RNA decoy, modulation of mRNA processing, DNA scaffolding, and by interacting with chromatin-modifying proteins (Hu et al., 2012; Wang and Chang, 2011).

Several lncRNAs have been associated with cell lineage determination and differentiation in adipocytes (Sun et al., 2013). Although *HOTAIR* has been described as an oncogene for various cancers (Bhan and Mandal, 2015), its physiological functions in *HOTAIR*-expressing tissues are largely unknown. In human fibroblasts, *HOTAIR* interacts with polycomb repressive complex 2 (PRC2) in the 5′ end, and lysine-specific histone demethylase (LSD1) in the 3′ end, to modulate H3K27 trimethylation and H3K4 demethylation, respectively (Tsai et al., 2010). The *HOTAIR/*PRC2/LSD1 complex can collaboratively suppress target genes in *trans*, such as genes in the *HOXD* locus on chromosome 2 (Rinn et al., 2007; Tsai et al., 2010). Intriguingly, the *HOTAIR* gene is located within a human fat distribution genome-wide association study (GWAS) locus (*HOXC13*) (Shungin et al., 2015). There is also evidence that *HOTAIR* expression is regulated by hormones implicated in body fat patterning such as estradiol (Bhan et al., 2013; Wells, 2007). Taken together, we hypothesized that *HOTAIR* is functionally important in regional AT development and may be essential for maintaining regional AT epigenetic cellular memory (defined as tissue-specific differences in transcriptional profile, mediated by chromatin modifications, which determine cell identity and function).

Here, we have studied the tissue-specific regulation of *HOTAIR* expression, modulated its expression in relevant adipose cellular systems to observe the phenotypic consequences, and linked these data to large-scale genetic studies on human fat distribution.

## Results

### *HOTAIR* is exclusively expressed in GSAT and is regulated by body fat distribution-influencing hormones

**Figure 1:**
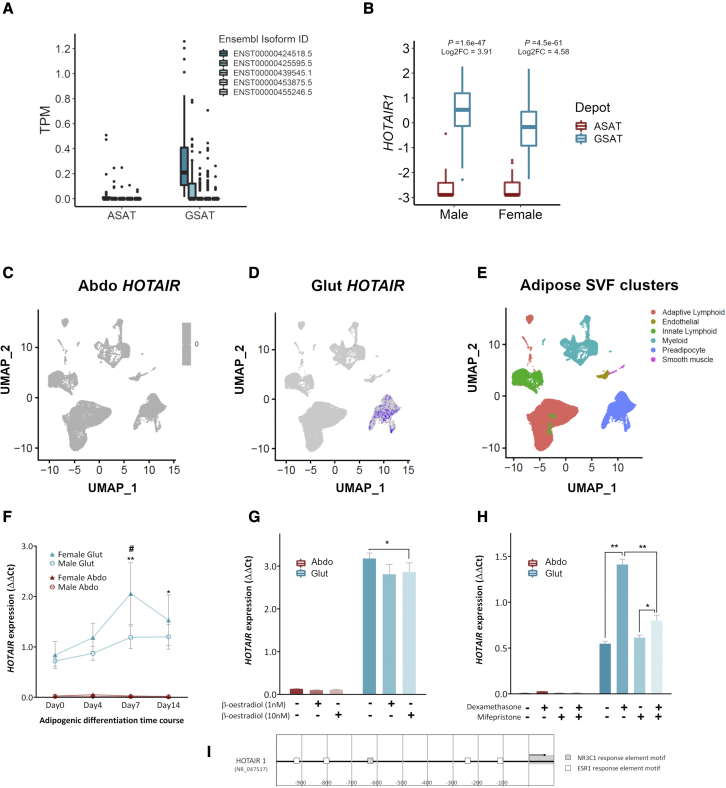
Tissue-specific expression of *HOTAIR* in gluteal AT (A) *HOTAIR* isoform abundance in paired ASAT and GSAT samples (61 females and 59 males; TPM, transcripts per million). (B) *HOTAIR1* expression in paired ASAT and GSAT samples (log2 counts per million). Statistical significance was assessed using (empirical Bayesian) modified t tests. Boxplots made in ggplot with outliers plotted as individual points. (C and D) Single-cell RNA-seq feature plots for *HOTAIR* expression in subpopulations of (C) ASAT stromovascular fraction (SVF) and (D) GSAT SVF. (E) Unsupervised clustering of AT SVF shown as uniform manifold approximation and projection (UMAP). (F) *HOTAIR* expression during adipogenesis in paired primary GSAT (Glut) and ASAT (Abdo) preadipocytes from females (triangles, n = 6) and males (circles, n = 10). Data presented as means ± SEMs. Statistical significance was assessed using 3-way ANOVA for time (p = 0.028), sex (p = 0.046), and depot (p < 0.001); ∗p < 0.05, ∗∗p < 0.001 (timepoint versus day 0); ^#^p < 0.05 ( male versus female). (G) *HOTAIR* expression in paired female primary GSAT (Glut) and ASAT (Abdo) preadipocytes following 24-h administration of β-estradiol (n = 6). (H) *HOTAIR* expression in _im_GSAT (Glut) and _im_ASAT (Abdo) following 48-h administration of dexamethasone (100 nM) and/or mifepristone (1 μM) (n = 3). Data for 1G and 1H are presented as means ± SEMs. Statistical significance was determined using 2-way ANOVA (with Tukey honestly significant difference [HSD] post hoc tests), ^∗^p < 0.05; ^∗∗^p < 0.001. (I) DNA-binding motifs for the estrogen receptor (ESR1) and glucocorticoid receptor (NR3C1) in 1-kb region upstream of *HOTAIR1*. n represents the number of experimental replicates.

*HOTAIR* is almost exclusively expressed in GSAT; few individuals show detectable *HOTAIR* expression in ASAT (4.14 _log_FC [fold change], p = 5.19 × 10^−70^; Figure 1A). Multiple isoforms are transcribed from the *HOTAIR* gene (Hajjari and Rahnama, 2017), and it is the long isoform (ENST00000424518.5; *HOTAIR1*) that is the most abundant in GSAT. Inter-individual variation in *HOTAIR1* expression in GSAT can be partly explained by sex differences. *HOTAIR1* is higher in males than females (0.72 _log_FC, p = 0.0004; Figure 1B), and, in males, *HOTAIR1* is positively associated with measures of lower-body fat (HIP_adjBMI_, Gynoid_adj.totalFat_) (Table 1). In recognition that *HOTAIR* expression is regulated by estrogen (Bhan et al., 2013; Milevskiy et al., 2016), and since the mean age of the female cohort was 49.2 years, we subdivided females into “premenopause, ≤49 years” and “peri-/postmenopause, ≥49 years” (Table 1). Positive associations with WHR and android visceral were observed in females 49 years and older, but there were no associations between *HOTAIR1* and measures of body fat in females 49 years and younger.

**Table 1:** Correlation coefficients between GSAT *HOTAIR* expression and anthropometric variables in OBB subjects

	*HOTAIR1* (ENST00000424518.5)					
	Male (n = 59)		Female age ≤49 y (n = 26)		Female age ≥49 y (n = 35)	
	Coeff.	p	Coeff.	p	Coeff.	p
Age _no correction_	−0.164	0.215	−0.134	0.515	0.321	0.060
BMI _no correction_	−0.034	0.797	0.208	0.308	0.242	0.162
WHR _no correction_	−0.233	0.076	0.251	0.216	**0.466**	**0.005**
Waist _adj. BMI_	−0.079	0.557	0.053	0.801	0.193	0.275
Hip _adj. BMI_	**0.279**	**0.034**	−0.148	0.480	−0.318	0.067
Gynoid _no correction_	0.131	0.323	0.014	0.944	0.012	0.944
Gynoid _adj. totalFat_	0.251	0.057	−0.263	0.204	−0.275	0.116
Android visceral _no. correction_	−0.111	0.401	0.268	0.186	0.307	0.073
Android visceral _adj. totalFat_	**−0.298**	**0.023**	0.191	0.360	**0.430**	**0.011**
Android subcut. _no. correction_	**0.281**	**0.032**	0.152	0.456	0.045	0.797
Android subcut. _adj. totalFat_	**0.354**	**0.006**	−0.064	0.761	−0.192	0.276

Data were assessed for normality by Shapiro-Wilk test. Spearman’s correlation or Pearson’s correlation were performed where appropriate. *HOTAIR1* expression was normally distributed in all groups. n represents the number of individuals in each group. Boldface type indicates statistical significance.

To provide a more comprehensive description of *HOTAIR* expression in AT, we performed a single-cell RNA sequencing (RNA-seq) analysis of the stromovascular fraction from GSAT and ASAT. This showed that *HOTAIR* expression is localized to the preadipocyte populations (Figures 1C–1E), and is not detected in the immune, endothelial, or smooth muscle cells, that are also resident in the tissue. Preadipocytes derived from GSAT and ASAT exhibit memory of *HOTAIR* expression in relation to their tissue-of-origin when cultured *ex vivo,* and these expression patterns are retained in differentiated adipocytes (Figure 1F, depot p < 0.001, 3-way ANOVA). *HOTAIR* expression in GSAT increases over time during adipogenesis (p = 0.028, 3-way ANOVA), with the highest expression observed at later time points on days 7 and 14 (Figure 1F). A sex effect was observed for *HOTAIR* expression in primary GSAT preadipocytes cultured *in vitro* (p = 0.046, 3-way ANOVA), which was opposite to that observed in whole AT (Figure 1B); female GSAT preadipocytes overall presented higher *HOTAIR* expression than male cells (Figure 1F).

We hypothesized that higher *HOTAIR* expression in female preadipocyte cultures may be due to the absence of estrogen in the culture system. A 1-kb region upstream of *HOTAIR1* was examined for hormone-binding motifs (JASPER CORE, 2018) (Khan et al., 2018) using the Eukaryotic Promoter Database (EPD) (https://epd.epfl.ch//index.php). DNA-binding motifs for the estrogen receptor (ESR1) and also the glucocorticoid receptor (GR) NR3C1 were identified (Figures 1G–1I). To test the effects of estrogen, confluent female primary cells were treated for 24 h with β-estradiol (1 or 10 nM). A modest reduction in *HOTAIR* expression in GSAT cells was observed at the higher dose (10 nM) (Figure 1G), suggesting that the absence of estrogen in the culture medium de-represses *HOTAIR* expression in female cultured cells. Glucocorticoids are also implicated in body fat patterning (e.g., chronic glucocorticoid excess, as seen in Cushing’s syndrome, is linked with central obesity) (Lee et al., 2014). Immortalized preadipocytes were treated with the synthetic glucocorticoid dexamethasone (100 nM) for 48 h and this selectively enhanced *HOTAIR* expression in immortalized GSAT (_im_GSAT) cells (Figure 1H). This effect was suppressed by co-administration of the GR antagonist, mifepristone, pointing to mediation via the glucocorticoid response element. Notably, *HOTAIR* expression in _im_ASAT cells did not respond to either estrogen or dexamethasone treatment and remained essentially undetectable. These findings suggest that tissue-specific responses to extrinsic modifiers, like steroid hormones, enable transcriptional fine-tuning of *HOTAIR* specifically in the gluteal depot.

### The *HOTAIR* gene locus is marked by tissue-specific regulatory histone modifications

**Figure 2:**
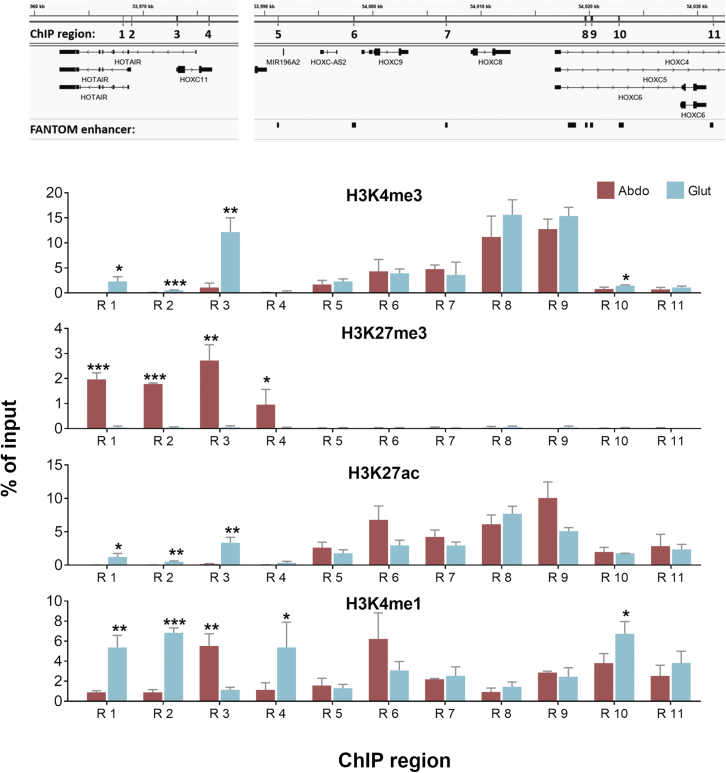
Tissue-specific histone modifications mark the promoter region of *HOTAIR* Chromatin immunoprecipitation (ChIP) analysis of histone modifications across the *HOTAIR* and *HOXC* locus in _im_GSAT (Glut) and _im_ASAT (Abdo) cells (n = 3). Data are shown as percentage of input (means + SEMs). Statistical significance was tested using Wilcoxon signed-rank test; ^∗^p < 0.05; ^∗∗^p < 0.01; ^∗∗∗^p < 0.001 (Abdo versus Glut). n represents the number of experimental replicates.

We have previously reported that tissue-of-origin memory for *HOTAIR* is retained as late as passage 30 in immortalized preadipocyte cell lines derived from ASAT and GSAT (Todorcevic et al., 2017), suggesting that the *HOTAIR* gene is subject to inherent, tissue-specific, epigenetic regulation in human AT. To address how tissue-of-origin *HOTAIR* expression patterns are maintained, we used chromatin immunoprecipitation (ChIP) to interrogate the state of regulatory histone modification marks across the *HOTAIR/HOXC* locus. Immortalized preadipocyte cell lines, referred to here as _im_GSAT and _im_ASAT (Todorcevic et al., 2017), were used to examine promoter regions (R1–R4), as well as putative enhancer elements (R5–R11) (Figure 2). The selected regions were examined for histone modifications most often associated with transcriptional activation (H3K4me3, near transcription start sites), transcriptional repression (H3K27me3, at promoters), enhancers (H3K4me1), and active enhancers (H3K27ac together with H3K4me1) (Shlyueva et al., 2014). Clear tissue-specific enrichments in these post-translational histone modifications were noted. Specifically, *HOTAIR* promoter regions (R1–R4) overall displayed marks consistent with higher *HOTAIR* expression in _im_GSAT cells relative to _im_ASAT cells, including H3K4me3, enhancer marking by H3K4me1, and active enhancer marking by H3K27ac (Figure 2), in line with the nearly undetectable *HOTAIR* expression in the abdominal cells. Accordingly, H3K27me3 strongly occupies the entire promoter region in _im_ASAT cells, indicating transcriptional repression (Figure 2). In contrast to the *HOTAIR* promoter regions, R5–R11, located in the *HOXC6-C9* loci, displayed similar histone modification profiles in _im_GSAT and _im_ASAT cells (Figure 2), suggesting that these putative enhancers do not contribute to tissue-of-origin differences in *HOTAIR* expression in these cell lines. Collectively, these data indicate that *HOTAIR* expression in preadipocytes is tightly controlled by differential histone modifications across the promoter, resulting in epigenetic inactivation of *HOTAIR* in _im_ASAT cells.

### *HOTAIR* interacts with the PRC2 complex in gluteal preadipocytes

**Figure 3:**
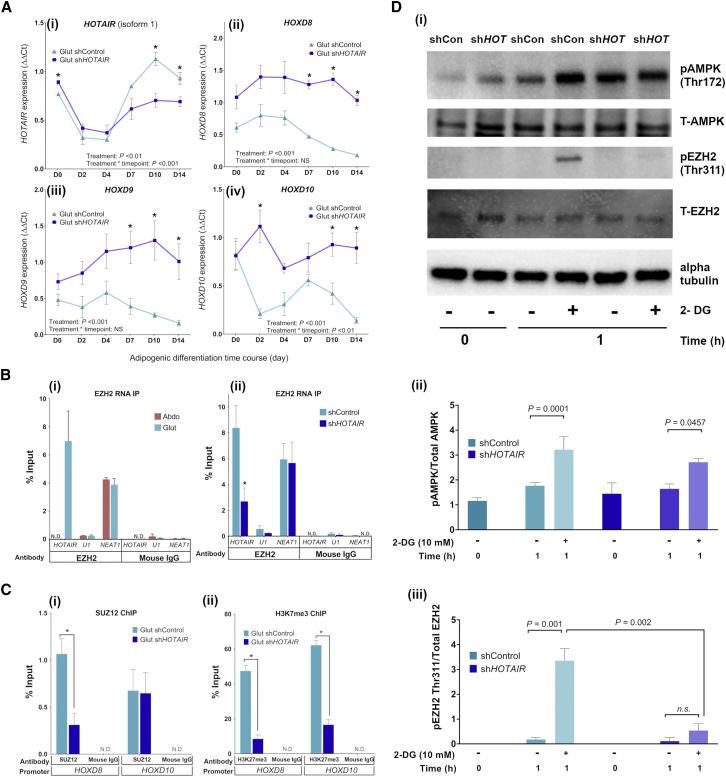
Functional interaction between *HOTAIR* and PRC2 in gluteal preadipocytes (A) Expression during adipogenesis of (i) *HOTAIR1*, (ii) *HOXD8*, (iii) *HOXD9*, and (iv) *HOXD10* in gluteal shControl and sh*HOTAIR* preadipocytes (n = 3). See also Figure S1. (B) RNA immunoprecipitation to assess interaction between EZH2 and *HOTAIR* or *NEAT1* at differentiation day 0 in confluent (i) _im_ASAT (Abdo) and _im_GSAT (Glut) cells (n = 3) and (ii) shControl and sh*HOTAIR* cells (n = 5). Immunoprecipitation with mouse immunoglobulin G (IgG) and absence of *U1* RNA were used as negative controls. See also Figures S2 and S3. (C) ChIP to assess (i) SUZ12 and (ii) H3K27me3 enrichment at the *HOXD8* and *HOX10* promoters at differentiation day 0 (n = 3). Immunoprecipitation with mouse IgG was used as a negative control. (D) Representative western blot (i) of phosphorylated (p) and total (T) AMPK and EZH2 in gluteal shControl and sh*HOTAIR* cells following 1-h treatment with 10 mM 2-deoxyglucose (2-DG) and in untreated cells (0 h). The ratios of (ii) pAMPK/total AMPK and (iii) pEZH2/total EZH2 were calculated (n=3). All data are presented as means ± SEMs. Statistical significance was assessed by 2-way ANOVA; ^∗^p < 0.05. n represents the number of experimental replicates.

To determine the role of *HOTAIR* in gluteal preadipocytes, a small hairpin RNA (shRNA) pool was used with the aim to ablate *HOTAIR* expression in _im_GSAT cells (referred to as sh*HOTAIR*). The shRNAs targeted regions in the final exon (nt 703–946) of *HOTAIR*. Expression of *HOTAIR1* was suppressed in sh*HOTAIR* cells, but this was only apparent after the day 4 time point (Figure 3A). Paradoxically, two short transcript variants (*HOTAIR2* and *HOTAIR3*) were upregulated in sh*HOTAIR* cells at later stages of adipogenesis (days 7, 10, and 14), despite sharing the final exon region targeted by the shRNA pool (Figure S1). Using a custom-designed TaqMan assay, a region overlapping 2 of the shRNA cleavage sites (nt 758–785) was successfully amplified in sh*HOTAIR* cells (Figure S1). The expression pattern of the amplified region mirrored that of *HOTAIR*2 and *HOTAIR*3, suggesting that RNA-induced silencing complex (RISC)-mediated cleavage of these transcript variants was not initiated. Together, these findings demonstrate a failure to successfully degrade all *HOTAIR* variants. This may be due to the high complexity of RNA secondary structure in the region targeted; all 4 shRNAs targeted RNA predicted to form base-pairing helices (Ameres et al., 2007; Somarowthu et al., 2015).

Given the conflicting *HOTAIR* mRNA expression in sh*HOTAIR* cells, downstream gene targets of *HOTAIR* were examined to determine whether its repressive function was impaired. *HOTAIR* serves as a molecular scaffold that tethers the histone modification complex, PRC2, to the *HOXD* locus where it coordinates H3K27me3 modification and transcriptional repression of *HOXD* genes (Rinn et al., 2007; Tsai et al., 2010). Several *HOXD* genes (*HOXD8*, *HOXD9*, *HOXD10*) displayed enhanced expression in sh*HOTAIR* cells (Figure 3A), which pointed to a loss of transcriptional repression of the *HOXD* locus in sh*HOTAIR* cells.

To examine the interaction between *HOTAIR* and PRC2, we performed RNA immunoprecipitation for the PRC2 subunit EZH2. *EZH2* displays higher expression than its homolog, *EZH1*, in _im_GSAT cells (Figure S2). Tissue-specific retrieval of *HOTAIR* was successfully demonstrated in _im_GSAT (*HOTAIR*^+^) and _im_ASAT cells (*HOTAIR*^−^) following the immunoprecipitation of EZH2 (Figure 3B). This was in keeping with the tissue-of-origin expression pattern of *HOTAIR*. Next, the interaction between *HOTAIR* and EZH2 was examined by EZH2 immunoprecipitation in sh*HOTAIR* cells. Retrieval of *HOTAIR* from the precipitate was ∼2-fold lower in sh*HOTAIR* cells compared to shControl cells (Figure 3B). Western blot analyses showed that EZH2 protein levels were lower in the nuclear lysate of sh*HOTAIR* cells despite no change in total cellular EZH2 protein (Figure S3). Taken together, this suggests that shRNA treatment of sh*HOTAIR* cells resulted in dysregulation of the interaction between *HOTAIR* and the PRC2 complex. Further support for this was provided by ChIP analyses, which revealed decreased occupancy of SUZ12 (another PRC2 subunit) at the promoter region of *HOXD8* in sh*HOTAIR* cells (Figure 3C). This was accompanied by a corresponding reduction in the PRC2-mediated histone mark, H3K27me3, across the same region (*HOXD8)*. H3K27me3 was also reduced across the *HOXD10* promoter (Figure 3C). Collectively, these findings support the loss of *HOTAIR*-PRC2 occupancy at the *HOXD* locus and indicate the disruption of *HOTAIR* interaction with PRC2 in sh*HOTAIR* cells.

lncRNAs are considered promiscuous in their interaction with PRC2 (Davidovich et al., 2013). To evaluate the specificity of *HOTAIR-*PRC2 binding, we measured another lncRNA, *NEAT1*, which also directly interacts with EZH2 (Wang et al., 2019). *NEAT1* was successfully retrieved in equal quantities in both _im_GSAT and _im_ASAT cells after EZH2 immunoprecipitation, and also in sh*HOTAIR* cells (Figure 3B), showing that the interaction between lncRNAs and PRC2 is not limited to *HOTAIR* in preadipocytes.

### *HOTAIR* is required for EZH2 interaction with AMPK

lncRNAs, such as *HOTAIR,* can act as biological scaffolds to aid the assembly of various protein and RNA complexes. To test the hypothesis that *HOTAIR* is required to support molecular interactions in gluteal cells, we investigated the interaction between EZH2 and the energy-sensing enzyme, AMP-activated protein kinase (AMPK). EZH2 is phosphorylated by AMPK on threonine 311 (Thr311), leading to the disassembly of PRC2 (Wan et al., 2018). Treating shControl and sh*HOTAIR* cells with 2-deoxyglucose resulted in AMPK activation in both sets of cells, as evidenced by phosphorylation of AMPK Thr172 (Figure 3D). In shControl cells, this was accompanied by the phosphorylation of EZH2 (Thr311). However, despite AMPK activation in sh*HOTAIR* cells, no phosphorylation of EZH2 (Thr311) was observed, suggesting that *HOTAIR* is involved in mediating crosstalk between cellular energy status and epigenetic regulation in gluteal preadipocytes.

### Loss of *HOTAIR*-PRC2 interaction in gluteal preadipocytes impairs adipogenesis

**Figure 4:**
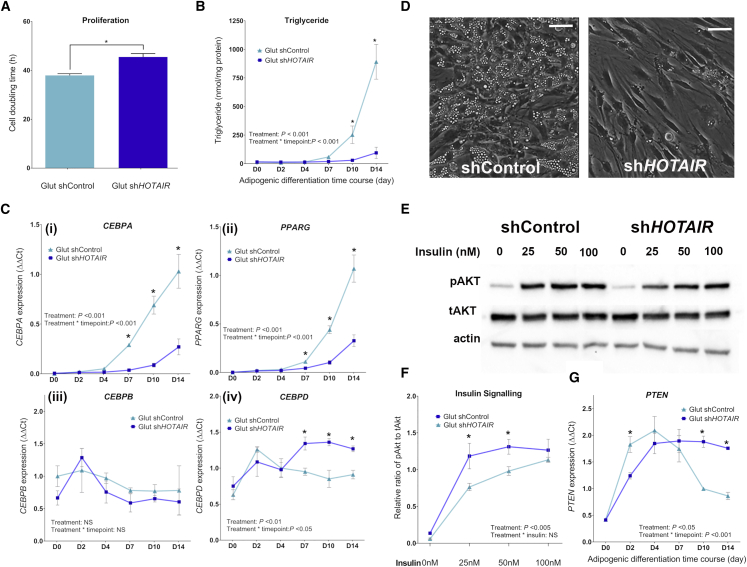
Effect of *HOTAIR* on preadipocyte proliferation and differentiation (A) Cell doubling time of gluteal shControl and sh*HOTAIR* cells (n = 4). (B) Intracellular triglyceride concentration normalized to total protein content (n = 3). (C) Expression of adipogenic transcription factors *CEBPA*, *PPARG2*, *CEBPB*, and *CEBPD* (n = 3). (D) Representative light microscopy images of cells at differentiation day 14 (×200 magnification, scale bar, 50 μm). (E) Representative western blot of phosphorylated (p) and total (t) Akt following 10-min insulin treatment on differentiation day 8. (F) Relative ratio of pAkt to tAkt (n = 3). (G) *PTEN* expression during adipogenesis (n = 3). Data presented as means ± SEMs. Statistical significance was assessed using 2-way ANOVA and/or independent 2-tailed t test; ^∗^p < 0.05; shControl versus sh*HOTAIR.* See also Figures S4 and S5. n represents the number of experimental replicates.

To investigate whether the interaction between *HOTAIR* and PRC2 plays a critical role in the development of gluteal adipocytes, we examined proliferation and adipogenesis. sh*HOTAIR* cells exhibited an extended cell doubling time compared to shControl cells, indicating a decreased proliferation rate (Figure 4A). During adipogenesis, the sh*HOTAIR* cells accumulated less triglycerides (Figure 4B) and maintained the spindle shape of preadipocytes, rather than acquiring a more rounded morphology with accumulation of lipid droplets, as was seen in the shControl cells (Figure 4D). Correspondingly, the late-stage “master” adipogenic transcription factors *CEBPA* and peroxisome proliferator-activated receptor gamma isoform-2 (*PPARG2*) were expressed at lower levels in sh*HOTAIR* cells from differentiation day 7 onward, whereas *CEBPD*, an early adipogenic marker, remained highly expressed during the late stages of adipogenesis (day 7 onward) (Figure 4C). In contrast, *CEBPB* expression was unaffected in sh*HOTAIR* cells. ChIP analysis indicated that the expression of *CEBPD* may be under epigenetic regulation by *HOTAIR*-PRC2 (Figure S4), with reduced occupancy of SUZ12 on the *CEBPD* promoter and a corresponding loss of the H3K27me3 mark in sh*HOTAIR* cells.

The phosphatidylinositol 3-kinase (PI3K)/Akt insulin signaling pathway is also a key regulator of proliferation and terminal adipocyte differentiation (Tomiyama et al., 1995). Insulin-stimulated phosphorylation of Akt on Ser473 (pAkt) was reduced in sh*HOTAIR* differentiated adipocytes compared to shControl (Figures 4E and 4F). Furthermore, the expression levels of phosphatase and tensin homolog (*PTEN*), a negative regulator of the PI3K/Akt pathway, remained elevated during late stages of adipogenesis in sh*HOTAIR* cells. This was contrary to the strong downregulation of *PTEN* observed between days 7 and 14 in shControl cells (Figure 4G). There was a trend for lower PRC2-mediated H3K27me3 at the *PTEN* promoter in sh*HOTAIR* cells, but SUZ12 occupancy was not reduced (Figure S4). Together, these data suggest that *PTEN* is not a direct target of *HOTAIR*-PRC2.

Our findings indicated that loss of *HOTAIR*-PRC2 interaction in gluteal cells impaired adipocyte development. Next, we questioned whether enhanced expression of *CEBPD* or *PTEN* was pathway limiting for adipogenesis. The expression of *CEBPD* or *PTEN* was repressed in sh*HOTAIR* cells using targeted small interfering RNAs (siRNAs) between differentiation days 4 and 14 (Figure S5). Suppression of *PTEN* in shControl cells led to a small increase in intracellular triglyceride content and expression of *CEBPA* and *PPARG*, compared with shControl cells, suggesting that *PTEN* suppression can improve adipogenic differentiation. However, suppressing *PTEN* in sh*HOTAIR* cells was not sufficient to rescue the impaired adipogenic phenotype of these cells; the triglyceride content remained 3-fold lower than in shControl cells. Similarly, lipid accumulation and adipogenic gene expression were not rescued by the suppression of *CEBPD* in sh*HOTAIR* cells between differentiation days 4 and 14 (Figure S5). Together, these findings implicate *HOTAIR*-PRC2 in the epigenetic regulation of key molecular regulators of adipogenesis but suggest that *CEBPD* and *PTEN* are unlikely to be pathway limiting alone, and that the adipogenic actions of *HOTAIR* extend beyond single-gene involvement.

### PRC2 target genes are de-repressed in sh*HOTAIR* cells

**Figure 5:**
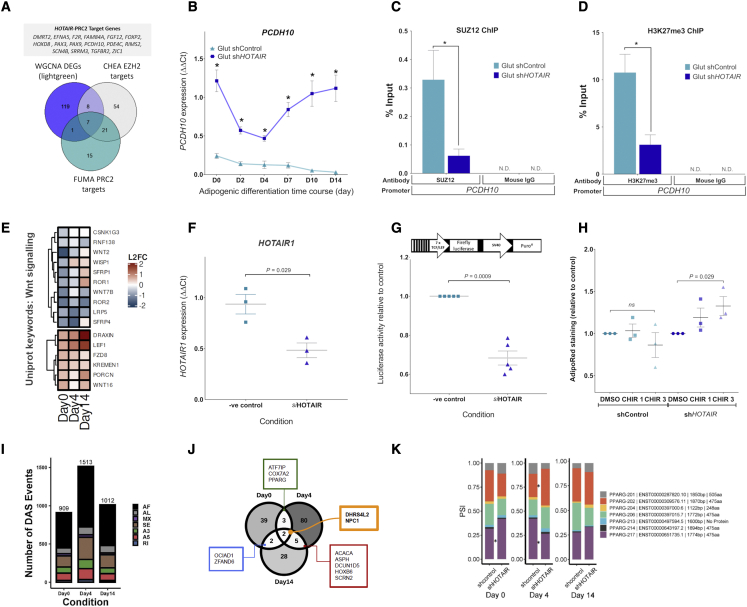
*HOTAIR-*PRC2 regulates developmental pathways in differentiating gluteal preadipocytes (A) Venn diagram showing overlap between PRC2 target genes (from FUMA and CHEA) and sh*HOTAIR* WGCNA DEGs (upregulated at all time points, light-green module). See also Figure S6. (B) qPCR confirmation of *PCDH10* expression in sh*HOTAIR* and shControl cells during adipogenesis (n = 3). Data were assessed using 2-way ANOVA; ^∗^p < 0.05. (C and D) ChIP to assess (C) SUZ12 and (D) H3K27me3 enrichment at the *PCDH10* promoter in gluteal sh*HOTAIR* and shControl cells on differentiation day 0 (n = 3). Mouse IgG antibody was used as a negative control. Data were assessed by Student’s t test; ^∗^p < 0.05. (E) Expression heatmap of sh*HOTAIR* DEGs annotated to UniProtKB: Wnt signaling (sh*HOTAIR* versus shControl; rlog normalized fold changes DESeq2). (F) *HOTAIR1* gene expression in _im_GSAT cells expressing the 7TFP TOPflash luciferase vector following 72 h *HOTAIR* siRNA treatment (n = 3, paired t test). (G) TOPflash promoter activity in Control and si*HOTAIR*_im_GSAT cells (n = 5, paired t test). (H) Adipogenesis assessed by AdipoRed staining in control and si*HOTAIR* cells treated with CHIR99021 1 μM and 3 μM throughout adipogenic differentiation (n = 3, ANOVA). (I) Differential alternative splicing (DAS) events between shControl and sh*HOTAIR* cells determined by SUPPA2:diffSplice. Alternative first (AF) or last (AL) exon, alternative 5′ or 3′ splice site (A5 and A3), mutually exclusive (MX) and skipped exons (SE), and retained introns (RI). (J) Number of genes that are differentially alternatively spliced at both the isoform (limma) and event (SUPPA2) level across each day of differentiation. (K) The proportion splice-in (PSI) of significantly expressed *PPARG* (GENCODE) isoforms in shControl and sh*HOTAIR* cells across each day of differentiation. ^∗^p < 0.05 (shControl versus sh*HOTAIR).* Data presented as means ± SEMs (B–D and F–H). See also Figure S7. n represents the number of experimental replicates.

RNA-seq was performed to profile the transcriptome of sh*HOTAIR* cells (Tables S1A–S1D). Because PRC2 is a transcriptional repressor, we began by examining differentially expressed genes (DEGs) that were upregulated in sh*HOTAIR* cells. We focused on day 0 (before broad transcript changes associated with impaired adipogenesis were apparent) and looked for overlap with curated gene sets from the Molecular Signatures Database (MSigDB). Using this unbiased approach, a strong overlap was seen with genes possessing H3K27me3 in their promoters or bound by the PRC2 subunits SUZ12 and EED (Meissner et al., 2008; Ben-Porath et al., 2008; Lee et al., 2006) (Figure S6; Table S2A). A subset of 44 genes was found to overlap with putative PRC2 targets identified in human embryonic stem cells (p = 1.4 × 10^−19^) (Ben-Porath et al., 2008). Upregulated day 0 DEGs were also independently examined for overlap with known EZH2 target genes annotated in the ChIP-X enrichment analysis (CHEA) database. Using this method, 90 overlapping genes were identified (p = 5.6 × 10^−15^, hypergeometric testing; Table S2B). Lastly, weighted correlation network analysis (WGCNA) identified 135 genes that were upregulated across all adipogenic time points in sh*HOTAIR* cells (days 0, 4, and 14; light-green module; Table S2C). Of those 135 genes, 16 were considered high-confidence PRC2 targets due to their overlap with the curated PRC2/EZH2 gene sets (Figure 5A). This core set of 16 genes represent putative *HOTAIR*-PRC2 targets that would normally be repressed during gluteal adipocyte development. They include the classical brown adipose marker *ZIC1* (Walden et al., 2012), the abdominal-specific transcript *DMRT2* (Pinnick et al., 2014; Karastergiou et al., 2013) and several lineage determination genes (*HOXD8*, *PAX3*, *PAX9*) (Bhatlekar et al., 2018; Blake and Ziman, 2014). For verification, we examined the recruitment of SUZ12 to the promoter of one of these core genes, the cadherin family member *PCDH10*, and observed reduced occupancy of SUZ12 in sh*HOTAIR* cells. This was accompanied by lower enrichment of H3K27me3 across the promoter and markedly higher expression of *PCDH10* across all time points (Figures 5B–5D). These findings highlight a role for *HOTAIR* and PRC2 in regulating lineage-specific genes in gluteal adipocytes.

### *HOTAIR*-PRC2 regulates growth factor/Wnt signaling in gluteal preadipocytes

Gene enrichment analysis was used to explore pathways differentially regulated in sh*HOTAIR* cells. The broad changes in sh*HOTAIR* transcriptional profile by day14 (Tables S1D–S2F) largely reflected the marked impairment of adipogenesis and proliferation, with downregulation of pathways relating to energy production and lipid metabolism (orange, Table S2E), and DNA replication and cell-cycle transition (yellow, Table S2E). We therefore focused on the smaller set of 619 day 0 DEGs (Table S1B) to identify early pathway changes present before phenotypic differences manifest. The most enriched Gene Ontology (GO) terms related to extracellular matrix organization and cell adhesion and included genes with conserved protein domains annotated to “epidermal growth factor-like domain,” “insulin-like growth factor binding protein, N-terminal,” and “cadherin” (INTERPRO, Table S2D). Proteins belonging to these families are typically membrane-bound or secreted peptides with roles in tyrosine kinase receptor signaling, DNA synthesis, and cell proliferation. Altered growth factor signaling is consistent with the extended doubling time exhibited by sh*HOTAIR* cells (Figure 4A).

Other notable GO terms included “canonical Wnt signaling pathway” and “skeletal system development” (Table S2D). As Wnt signaling modulates mesenchymal stem cell fate and adipocyte development (Bagchi and MacDougald, 2021; Christodoulides et al., 2009), we examined this pathway in more detail. Multiple members of canonical Wnt/β-catenin signaling were identified as differentially expressed (Figure 5E), including Wnts (*WNT2*, *WNT7B*, *WNT16*), Wnt receptors (*LRP5*, *KREMEN1*, *FZD8*), Wnt antagonists (*SFRP1*, *SFRP4*), and Wnt target genes (*WISP1*, *LEF1*). Next, canonical Wnt signaling activity was assessed in gluteal cells stably expressing the TOPflash promoter reporter construct (Fuerer and Nusse, 2010) following siRNA silencing of *HOTAIR* (si*HOTAIR*) (Figure 5F). Luciferase activity was significantly lower in si*HOTAIR* cells compared to controls, indicating impaired Wnt signaling (Figure 5G). To test this further, sh*HOTAIR* and shControl cells were treated with a chemical Wnt signaling activator (CHIR99021) throughout adipogenesis (Figure 5H). Treatment with CHIR99021 (3 μM) selectively enhanced adipogenesis in sh*HOTAIR* cells (assessed by lipid staining) by ∼25% relative to vehicle. However, despite this modest gain of function, absolute lipid levels remained ∼4 times lower than shControls (81,315 ± 13,531 versus 21,429 ± 2,396 a.u., p < 0.0001). These data indicate that the dysregulation of Wnt signaling in sh*HOTAIR* preadipocytes may contribute to the failure of these cells to undergo adipogenesis; however, the activation of canonical Wnt signaling is not sufficient to fully reverse the phenotype.

### sh*HOTAIR* cells exhibit genome-wide alternative splicing events

In addition to PRC2, *HOTAIR* is known to interact with splicing factor proteins such as heterogeneous nuclear ribonucleoprotein (hnRNP) A2/B1 (Nguyen et al., 2018) to regulate alternative splicing (Pisignano and Ladomery, 2021). Given the central role of alternative splicing during development and tissue differentiation (Mazin et al., 2021), the sh*HOTAIR* RNA-seq data was analyzed to test the hypothesis that *HOTAIR* regulates alternative splicing in GSAT. Isoforms exhibiting differential transcript usage (Table S3A) and differentially regulated splicing events (Figure 5I) were detected between sh*HOTAIR* and shControl cells at all time points. The most frequent differentially regulated splicing events were alternative first exon (AF) and skipped exons (SE) (Figure 5I). Differential alternative splicing disproportionately occurred at genes linked to RNA, protein, and heterocyclic compound binding, as well as pathways related to metabolism (“fatty acid biosynthesis”) and apoptosis (“programmed cell death”) (Figure S7). A subset of high-confidence genes (Figure 5J) was found to be differentially alternatively spliced by both the isoform- and event-level methodologies across multiple days, including *ACACA*, the rate-limiting step of fatty-acid biosynthesis, and the master regulator of adipogenesis, *PPARG.* In particular, sh*HOTAIR* cells exhibited altered inclusion of the canonical *PPARG* isoform 217 (ENST00000651735.1; *PPARG3*) at day 0 and both PPARG-217 and PPARG-202 (ENST00000309576.11; *PPARG1*) at day 4 compared to shControl cells. These data implicate *HOTAIR* in the widespread regulation of GSAT isoform diversity, the dysregulation of which may be a further factor contributing to unsuccessful adipogenesis in sh*HOTAIR* preadipocytes.

### Identification of an independent *HOTAIR* expression quantitative trait locus (eQTL) associated with human body fat distribution

**Figure 6:**
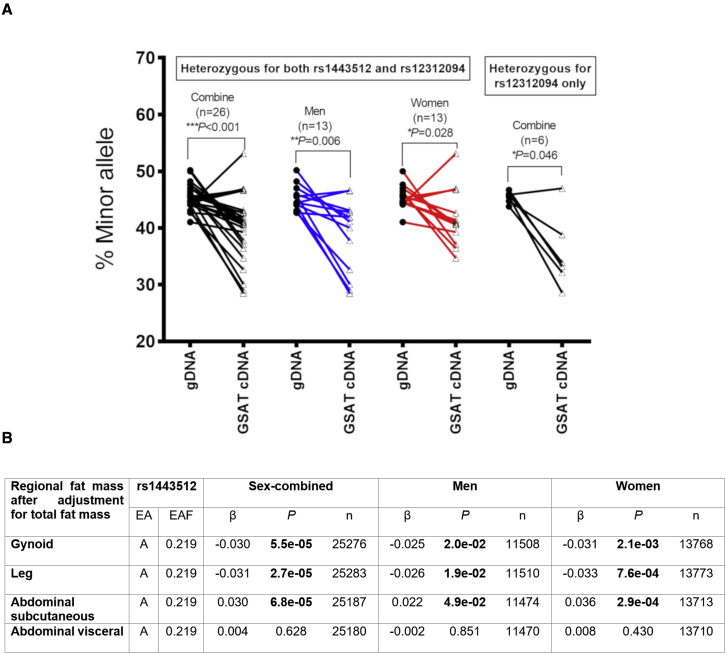
An independent *HOTAIR* eQTL is associated with human body fat distribution (A) Allelic expression of the *HOTAIR* transcript assessed by allele-specific qPCR in GSAT cDNA samples from 26 carriers heterozygous for the rs1443512-rs12312094 haplotype and 6 heterozygous for rs12312094 alone. Genomic DNA (gDNA) from the same individuals were used as paired controls with presumed equal allele expression. Data were assessed using Wilcoxon signed-rank test; ^∗^p < 0.05; ^∗∗^p < 0.01; ^∗∗∗^p < 0.001. (B) Associations between DXA-defined regional fat mass and rs1443512 genotypes. EA, effect allele; EAF, effect allele frequency. p values and β-coefficients for associations with DXA-defined regional fat mass after adjustment for total fat mass.

The *in vitro* findings identified *HOTAIR-*PRC2 as a regulator of regional gluteal adipogenesis. Next, we investigated whether genetic variation across the *HOTAIR* locus influences adipose phenotypes. A previous GWAS identified 3 signals (A: rs1443512, B: rs10783615, and C: rs2071449) associated with body fat distribution within the *HOXC* locus (Shungin et al., 2015). Two of the signals (rs1443512 and rs10783615) are in linkage disequilibrium (LD; D′ 0.99, r^2^ 0.55), and displayed weak eQTL effects for *HOTAIR1* in GSAT (rs1443512: p = 0.046, β = −0.179; rs10783615: p = 0.021, β = −0.21; linear regression adjusted for sex), suggesting the minor (WHR increasing) allele was associated with lower *HOTAIR1* expression. To validate the *HOTAIR* eQTL effect, an SNP in the last exon of *HOTAIR* (rs12312094, MAF 0.03), and in moderate LD with rs1443512 (D′ 0.56), was selected for allele-specific qPCR (Neville et al., 2019) (Figure 6A). In individuals heterozygous for both SNPs on the same haplotype (imputed allele frequency 0.16), the minor (WHR increasing) allele displayed lower *HOTAIR* expression in GSAT (Figure 6A). However, 6 individuals heterozygous for rs12312094 alone also exhibited the same allelic imbalance (Figure 6A). This indicates that the eQTL effect is not caused by rs1443512, but by another variant on the more common haplotype containing rs12312094, which requires future investigation. In summary, these findings show that common genetic variation in the *HOXC* GWAS locus influences *HOTAIR* expression.

To bring further clarity to the regional AT depots influenced by the *HOTAIR* eQTL, we analyzed the relationship between rs1443512 and DXA measurements of fat mass in a meta-analysis of 25,276 individuals. In contrast to the earlier GWAS, which used WHR (Shungin et al., 2015), the DXA measurements provide defined tissue-specific fat masses allowing further investigation of the genetic association (Figure 6B). Leg and gynoid fat mass were both lower in carriers of the minor allele of the rs1443512 eQTL, whereas an opposite effect was observed for android fat mass (Figure 6B). This is consistent with the minor allele lowering *HOTAIR* expression and exerting an anti-adipogenic effect in leg and gynoid. A likely explanation for the reciprocal increase in upper-body fat mass is a compensatory redistribution of fat storage due to the restriction imposed in the lower body. The effect on the android region was confined to ASAT, with no association seen for visceral fat mass. This is reasonable, given that upper-body subcutaneous fat is the largest AT compartment in the human body. Together, these findings provide genetic evidence that a certain degree of *HOTAIR* expression is required for the development of gluteal AT.

## Discussion

Using human cells and tissues derived from ASAT and GSAT, we explored the functional role of *HOTAIR* in human body fat distribution and provide evidence that *HOTAIR* interacts with PRC2 and modulates regional adipocyte development. To date, at least 10 lncRNAs, including *HOTAIR*, have been implicated in the regulation of human white adipocyte differentiation and function (Sun and Lin, 2019). However, findings for *HOTAIR* have not always been consistent; *HOTAIR* negatively regulated the differentiation of mesenchymal stem cells toward both adipogenic and osteogenic lineages (Kalwa et al., 2016), whereas ectopic overexpression of *HOTAIR* in human abdominal preadipocytes was reported to promote adipogenesis (Divoux et al., 2014). Our findings suggest that the actions of *HOTAIR* are tissue context dependent. We have recently reported anti-adipogenic effects when *HOTAIR* is artificially expressed in abdominal preadipocytes (Kuo et al., 2022), and here, we show pro-adipogenic effects in gluteal cells, where it is naturally expressed. The opposing actions on adipocyte development are in keeping with the striking differences in *HOTAIR* expression between ASAT and GSAT, and this highlights the value of studying multiple AT depots when examining the regulation of body fat distribution.

The mechanisms by which lncRNAs act are diverse (Wang and Chang, 2011). We focused on the interaction between *HOTAIR* and the chromatin-modifying complex PRC2 in developing adipocytes. The PRC2 subunit EZH2 (and related paralog EZH1) mediate repressive H3K27me3 over broad regions across the genome (Laugesen et al., 2019). Scaffolding lncRNA, such as *HOTAIR*, likely act as focal points to direct PRC2 to target sites, and this requires RNA-targeting motifs for site-specific tethering (Chu et al., 2011). In line with this, we found that *HOTAIR* interacts with EZH2 in gluteal cells, and we observed a loss of H3K27me3 on target promoters in sh*HOTAIR* cells. As EZH2 shares functional redundancy with EZH1 (Wassef et al., 2019), future evaluation of *HOTAIR-*EZH1 interactions is needed. Questions have been raised regarding the functional significance of *HOTAIR*-PRC2 interactions (Brockdorff, 2013). PRC2 is not required for *HOTAIR* to tether to chromatin (Chu et al., 2011), and *HOTAIR*-mediated transcriptional repression has been reported in PRC2-depleted breast cancer cells (Portoso et al., 2017). Thus, PRC2 recruitment may be a secondary event in transcriptional regulation. We found that shRNA targeting *HOTAIR* disrupted the interaction with PRC2 in gluteal cells (despite detectable *HOTAIR* mRNA remaining). The involvement of *HOTAIR* in recruiting PRC2 to target genes in gluteal cells is supported by the loss of SUZ12 occupancy on the promoters of *HOXD8* and *PCDH10*, and by a reduction in H3K27me3. Nuclear EZH2 protein levels were lower in sh*HOTAIR* cells despite no change in whole-cell EZH2 protein levels, suggesting the displacement of PRC2 from the nucleus. Since PRC2 subunits exist in stoichiometric balance, this also accounts for the lower occupancy of SUZ12 on target promoters. *HOTAIR* is not unique in its interaction with PRC2, which has high affinity but low specificity for RNA binding (Davidovich et al., 2013). Other PRC2-interacting lncRNAs include *XIST* and *NEAT1* (Davidovich et al., 2013). We also noted the loss of EZH2 protein phosphorylation in sh*HOTAIR* cells under conditions of energy restriction. Negative regulation of EZH2 by AMPK allows crosstalk between cellular energy status and epigenetic modification (Wan et al., 2018). We propose that *HOTAIR* may be required as a scaffold to permit this signaling in gluteal adipocytes. In times of negative energy balance, this would limit the formation of new adipocytes through the dysregulation of the PRC2 complex.

Cell proliferation and adipogenesis were impaired in the sh*HOTAIR* cells. *HOTAIR* overexpression in endometrial cancer promotes proliferation by direct inhibition of the tumor suppressor PTEN and activation of the PI3K/Akt pathway (Zhang et al., 2019). In sh*HOTAIR* cells, *PTEN* was upregulated and insulin signaling was impaired, which may contribute to the lower rate of proliferation. Interestingly, lower-body (*HOTAIR*^+^) AT depots display a proliferative response to weight gain in adults, whereas abdominal (*HOTAIR*^−^) AT expands by hypertrophy (Tchoukalova et al., 2010). Hyperplastic AT expansion has been associated with improved insulin sensitivity (Arner et al., 2010); thus, *HOTAIR* may contribute to improved whole-body metabolic regulation through the generation of new adipocytes in GSAT. In ASAT, the absence of *HOTAIR* may be a limiting factor for AT expansion, leaving the tissue reliant on hypertrophic expansion. We noted a positive association between gluteal *HOTAIR* expression and measurements of lower-body AT in males, and this was directionally consistent with the *HOTAIR* eQTL (rs1443512), where the WHR-increasing allele was associated with lower *HOTAIR* expression and reduced leg and gynoid fat mass.

The failure to undergo adipogenesis in sh*HOTAIR* cells is not absolute but is consistent with the severe defect in adipogenesis seen in *Ezh2*^*−/−*^ white preadipocytes (Wang et al., 2010). That defect was attributed to the loss of H3K27me3 and de-repression of Wnt genes (Wang et al., 2010). In sh*HOTAIR* cells, genes annotated to Wnt signaling were differentially expressed, including *LRP5*, which promotes lower-body fat distribution (Loh et al., 2015a). We show that the depletion of *HOTAIR* in gluteal cells impairs Wnt signaling and that adipogenesis can be partially rescued by activating canonical Wnt signaling. Although typically considered an anti-adipogenic pathway, we have previously reported a positive relationship between Wnt activity and adipogenesis specifically in gluteal-derived adipocytes (Loh et al., 2015a). These findings highlight the complexity of canonical Wnt signaling, which can exert distinct actions on cell function, depending on tissue and stage of development (Bagchi and MacDougald, 2021).

Gene expression analysis identified hundreds of DEGs in sh*HOTAIR* cells, including a high-confidence set of 16 *HOTAIR-*PRC2 target genes. Some of these genes are lineage determining (*DMRT2*, *HOXD8*, *PAX3*, *PAX9)* and would typically be repressed during gluteal adipogenesis. There were also considerable changes in alternative splicing events, including *PPARG*. Alternative splicing of *PPARG* generates multiple transcript variants but only 2 protein isoforms (Aprile et al., 2014; Hernandez-Quiles et al., 2021). The transcript-level changes we report in sh*HOTAIR* cells relate to *PPARG1* and *PPARG3*, which differ in their 5′ UTR and pattern of expression during adipogenesis, while both give rise to the PPARγ1 protein (475 amino acids [aa]). The functional significance of alternative splicing of *PPARG* remains unclear, but as a master regulator of adipogenesis, this may be a further factor contributing to the unsuccessful adipogenesis of sh*HOTAIR* preadipocytes. Overall, the RNA-seq analysis illustrates the global role that *HOTAIR*-PRC2 plays in regulating the diversity of the preadipocyte transcriptome and proteome. This is further highlighted by the finding that adipogenesis could not be rescued by single-gene manipulation *(PTEN*, *CEBPD*).

Preadipocytes possess an intrinsic tissue-of-origin memory (Macotela et al., 2012; Pinnick et al., 2014; Tchkonia et al., 2007) and retain transcriptional profiles when cultured *ex vivo,* pointing to the involvement of epigenetic regulation. ASAT and GSAT display differential DNA methylation patterns (Gehrke et al., 2013; Pinnick et al., 2014), and assay for transposase-accessible chromatin with high-throughput sequencing (ATAC-seq) has identified differences in open chromatin between the 2 tissues (Divoux et al., 2018). In agreement, we report marked enrichments of tissue-specific histone modifications across the *HOTAIR* promoter regions. In gluteal cells, where histone marks indicate open chromatin (H3K4me3, H3K27ac), *HOTAIR* expression is also subject to fine-tuning by hormones with known AT redistribution effects (estrogen and glucocorticoids). The positive association observed between *HOTAIR* expression and visceral fat mass in females 49 years and older may reflect the de-repression of *HOTAIR* expression due to declining estrogen levels and an independent (menopause-associated) increase in visceral fat mass in these individuals (Dehghan et al., 2021). Thus, factors influencing estrogen levels may be confounding when examining *HOTAIR* expression in females, and this could explain some of the heterogeneity in GSAT *HOTAIR* expression.

In conclusion, we demonstrate that depot-specific *HOTAIR* expression is functionally important in the regulation and development of regional AT and is specifically required for maintaining adequate biological function of the gluteal AT depot. One of the most paradoxical findings from this study is that *HOTAIR* is essentially undetectable in ASAT, and yet this depot also possesses the capacity for adipogenic differentiation. Thus, it would seem that there are fundamental differences in the regulation of adipogenesis between ASAT and GSAT. Identifying differentially expressed lncRNA that associate with chromatin-modifying complexes in gluteal and abdominal preadipocytes is of paramount importance to understanding differences in how these AT depots develop.

### Limitations of the study

A limitation of this study is that we could not consistently demonstrate a lowering of *HOTAIR* transcript levels in the shRNA experiments, although there were clear functional consequences consistent with impaired *HOTAIR* function. Some lncRNA are reportedly difficult to suppress by siRNA techniques, especially those that, like *HOTAIR*, also localize to the nucleus (Lennox and Behlke, 2016). We are not the first group to report the effects of a functional lncRNA knockdown by interfering with functional domains without initiating degradation of the target transcript (Sarma et al., 2010; Ulitsky et al., 2011). That the interaction between *HOTAIR* and PRC2 was disrupted in the *in vitro* sh*HOTAIR* experiments is supported by the lower retrieval of *HOTAIR* following EZH2 RNA immunoprecipitation. Furthermore, the human *HOTAIR*-lowering eQTL data can be viewed as a natural *in vivo* knockdown, and this was associated with reduced gluteal fat mass consistent with the adipogenic defect seen in the gluteal sh*HOTAIR* cells.

## STAR★Methods

### Key resources table

**Table:** 

REAGENT or RESOURCE	SOURCE	IDENTIFIER
**Antibodies**		

anti-phosphorylated Akt	Cell Signalling	#4060; RRID:AB_2315049
anti-total Akt	Cell Signalling	#4685; RRID:AB_2225340
anti-AMPKa	Cell Signalling	#5831; RRID:AB_10622186
anti-pAMPK Thr172	Cell Signalling	#2535; RRID:AB_331250
anti-EZH2	Cell Signalling	#5246; RRID:AB_10694683
anti-pEZH2 Thr311	Cell Signalling	#27888; RRID:AB_2798950
anti-α-tubulin	Abcam	ab15246; RRID:AB_301787
goat anti-rabbit IgG	Agilent Dako	#P0448; RRID:AB_2617138
goat anti-rabbit IgG (H+L)	Invitrogen	#31460; RRID:AB_228341
conjugated anti-actin (I-19) HRP	Santa Cruz Biotechnology	sc-1616 HRP; RRID:AB_630836
anti-EZH2; clone AC22	Millipore	Kit 03-900; Part no. CS203195
Mouse IgG	Millipore	Kit 03-900; Part no. CS200621
anti- H3K4me3	Diagenode	C15410003; RRID:AB_2616052
anti- H3K27me3	Diagenode	C15410069; RRID:AB_2814977
anti- H3K27me3	Abcam	ab6002; RRID:AB_305237
anti- H3K27ac	Diagenode	C15410174; RRID:AB_2716835
anti- H3K4me1	Diagenode	C15410037;RRID:AB_2561054
anti -SUZ12	Abcam	ab12073; RRID:AB_442939

**Bacterial and virus strains**		

HOTAIR piLenti-siRNA-GFP	Applied Biological Materials (abm)	iv009919
Scrambled piLenti-siRNA-GFP	Applied Biological Materials (abm)	LVP015-G

**Chemicals, peptides, and recombinant proteins**		

β-oestradiol	Sigma	E2758
Mifepristone	Sigma	M8046
2-Deoxy-D-glucose	Sigma	D8375
CHIR99021	Abcam	ab120890

**Critical commercial assays**		

Luciferase Assay System	Promega	E1500
Triacylglycerol assay	Instrumentation Laboratories	N/A
AdipoRed assay	Lonza	PT-7009
DC protein assay	Bio-Rad	5000112

**Deposited data**		

HOTAIR shRNA RNAseq data	GEO	GEO: GSE205350
Raw Western blots	Mendeley Data https://data.mendeley.com/	Mendeley Data: https://doi.org/10.17632/jxy5g24t9g.1

**Experimental models: Cell lines**		

Immortalised human preadipocytes	N/A	Todorcevic et al. 2017

**Oligonucleotides**		

See Table S4 for Taqman gene expression assays	ThermoFisher Scientific	N/A
See Table S5 for ChIP primers used to assess histone modification marks	Bio-Rad ThermoFisher Scientific	N/A
See Table S6 for custom designed Taqman assays for gene promoter regions	Bio-Rad ThermoFisher Scientific	N/A
siRNA CEBPD	Origene	SR300761
siRNA PTEN	Origene	SR321496
The siRNA HOTAIR pool was custom designed using the target sequences: GAGGAAAAGGG AAAATCTA; GAACGGGAGTACAGAGAGA; CCACATGAACGCCCAGAGA; TAACAAGACCAGAGAGCTG;	Dharmacon siRNA custom tool	N/A
Lincode Non-targeting negative control siRNA	Dharmacon	N/A

**Software and algorithms**		

Prism 8	GraphPad Software, Inc	https://www.graphpad.com
SPSS	IBM	https://www.ibm.com/analytics/spss-statistics-software
Encore software for DXA (version 11.0)	GE. Medical Systems	N/A
DAVID Bioinformatics Resources	https://david.ncifcrf.gov/home.jsp	PMID: **35325185;** https://doi.org/10.1093/nar/gkac194
FUMA GWAS GENE2FUNC	https://fuma.ctglab.nl/	PMID: **29184056;** PMCID: PMC5705698; https://doi.org/10.1038/s41467-017-01261-5;
ChIP-X Enrichment Analysis Resource	https://maayanlab.cloud/Harmonizome/resource/ChIP-X+Enrichment+Analysis	PMID: **20709693;** PMCID: PMC2944209; https://doi.org/10.1093/bioinformatics/btq466;

### Resource availability

#### Lead contact

Further information and requests for resources and reagents should be directed to and will be fulfilled by the lead contact Katherine Pinnick (katherine.pinnick@ocdem.ox.ac.uk).

#### Materials availability

This study did not generate new unique reagents.

### Experimental model and subject details

#### Participant recruitment from Oxford Biobank (OBB)

The Oxford Biobank is a population-based repository containing biological samples and phenotype information on approx. 8000 healthy participants recruited between the ages of 30–50 years of age from Oxfordshire, UK (Karpe et al., 2018). All participants gave signed informed consent. Ethical approval was granted by Oxfordshire Clinical Research Ethics Committee (WAT study: 08/H0606/107). For whole AT RNA-sequencing, AT biopsies were obtained from 120 healthy participants (59 male, 61 female) with average age 49.2 years ±7.03 (SD) and average BMI 26.3 kg/m^2^ ± 1.5 (SD). For each sex, recruitment was stratified to ensure equal numbers of lean/obese and an equal distribution of WHR within each lean/obese group. Anthropometric measures were collected, and body composition was assessed by DXA (GE Lunar iDXA) with Encore software (version 11.0; GE. Medical Systems, Madison, WI, USA), on the day of the AT biopsy. For single cell RNA-seq (ScRNA-seq), AT biopsies were obtained from 32 healthy participants (15 male, 17 female) with average age 46.8 years ±5.0 (SD) and average BMI 25.5 kg/m^2^ ± 1.5 (SD). For allele-specific qPCR, AT cDNA and genomic DNA was obtained from 26 healthy participants heterozygous for both rs1443512 and rs12312094 (13 male, 13 female) and 6 healthy participants who were heterozygous for rs12312094 only (4 male, 2 female).

#### Human primary preadipocytes

Human primary preadipocytes were isolated from ASAT and GSAT biopsies (Hauner et al., 2001; Collins et al., 2010) from participants of the Oxford Biobank. For the adipogenesis time-course experiment, primary cells were prepared from 16 participants (10 male, 6 female). For the β-oestradiol treatment experiment, primary cells were prepared from 6 female participants.

#### Human immortalised preadipocyte cell lines

The human immortalised preadipocyte cell lines used in this study were _im_ASAT/_im_GSAT cells. These cell lines have been described in more detail in (Todorcevic et al., 2017). Briefly, the cell lines were derived from a pair of AT biopsies collected from the ASAT and GSAT depots of a 50 year old, healthy, male participant of the Oxford Biobank (BMI: 24.4 kg/m2). The cell lines were generated by co-expression of the human telomerase (hTERT) gene and the human papillomavirus type-16 E7 oncoprotein (HPV16-E7).

#### Genetic association cohorts

A meta-analysis of 25,276 individuals was performed to assess the relationship between the rs1443512 SNP and DXA measurements of fat mass. The participants were obtained from the Fenland study (n = 10,309) (Lotta et al., 2016), EPIC-Norfolk (n = 4,440) (Day et al., 1999), UK Biobank (n = 4,707) (Miller et al., 2016) (Bycroft et al., 2017), Oxford Biobank (n = 4,572) (Karpe et al., 2018), and ORCADES (n = 1,979) (McQuillan et al., 2008). Included in the final analyses were approx. 11, 500 males and 13, 700 females. All studies were approved by the local ethics committees and participants gave their written informed consent prior to entering the study. Details of participant recruitment, DXA body composition analysis and genotyping for Fenland, EPIC-Norfolk, Oxford Biobank and UK Biobank have previously been described (Lotta et al., 2017) (Neville et al., 2019) (Lotta et al., 2018). In ORCADES, participants underwent whole-body DEXA scans using the Hologic QDR4500 densitometer machine and images were processed using the manufacturer’s APEX4 software. Fat and lean mass measures >5 SDs from the mean were omitted. Genome-wide genotyping in ORCADES was done in two phases. Panel A consisted of 890 participants and was genotyped using the Illumina HumanHap300v2. The Illumina OmniX or Omni1 were used to genotype panel B which was comprised of 1,300 participants. Only variants represented on both the OmniX and Omni1 array were retained for analyses. SNPs were omitted if the call rate <98%, MAF <0.01 or Hardy-Weinberg equilibrium p-value <10^−6^. Duplicate samples were removed, and individuals of non-European ancestry were identified and excluded based on a scatter plot of the first two genetic principal components anchored in the 1000G phase 3 CEU, YRI and CHB/JPT reference panels. Imputation to the HRC reference panel was done for panels A and B separately.

### Method details

#### Collection and processing of human AT samples for RNAseq platforms

Paired AT biopsies were taken under local anaesthetic (1% lignocaine) by needle aspiration at the level of the umbilicus (ASAT) and from the upper-, outer-quadrant of the gluteal region (GSAT).

For whole AT RNA-sequencing, AT samples (approx. 200mg) were washed in saline and stored in RNAlater at – 80°C. Total RNA was extracted using TRI-Reagent. cDNA libraries were generated by Novogene (Cambridge Science Park) following rRNA-depletion and samples were sequenced on an Illumina NovaSeq 6000 PE150 platform. Trimmed sequencing reads were pseudo-aligned to the human transcriptome (GENCODE GRCh37 hg19) using salmon (Patro et al., 2017) before being imported into RStudio at both the gene- and isoform-levels with TXimport (Soneson et al., 2015). Lowly expressed features were removed using the edgeR filterByExpr function before being normalised using the upper-quartile method. The limma (Ritchie et al., 2015) voomWithQualityWeights pipeline was used to identify differentially expressed genes and transcript isoforms between abdominal and gluteal depots whilst accounting for sample pairings using the duplicateCorrelations method and correcting for sequencing batch effect and participant age.

For ScRNA-seq, AT samples were homogenised, collagenase digested and centrifuged to isolate the stromovascular fraction (SVF). SVF cells were re-suspended in FBS containing 10% DMSO and stored in liquid nitrogen. ScRNA-seq libraries were generated using 10x Genomics Chromium Single Cell 3′ Library & Gel Bead and i7 Multiplex kits. Sequencing was performed on an Illumina NextSeq500 using a 75-bp paired-end kit. Following alignment, and demultiplexing using Cell Ranger (10x Genomics) and demuxlet (Kang et al., 2018), the expression matrix was generated. The R package Seurat (https://satijalab.org/seurat) was used for quality control, data normalization and scaling, clustering and annotation of cell types. The data were obtained from a 10x scRNA-seq dataset containing 54708 subcutaneous abdominal and gluteal stromal vascular cells. Here, we show only the look-up for *HOTAIR* to confirm cellular distribution of gene expression, the rest of the data are unpublished.

#### Culture and differentiation of human primary or immortalised preadipocyte

Human preadipocytes were cultured in growth medium until confluent then treated with adipogenic medium for 14 days (Todorcevic et al., 2017). For proliferation studies cells were seeded at a density of 2 ×10^5^ cells into 75cm^2^ flasks and cultured for 4 days at which point the cells were still sub-confluent. Cells were counted using a Cellometer Auto T4 (Nexcelom Bioscience). Doubling time (DT) was calculated using the following formula: DT = T × ln2/ln(cells^end^/cells^start^) where T = culture time (hours) (Loh et al., 2015a). For hormone treatment experiments confluent cells were treated with β-oestradiol (1nM, 10nM) for 24 h or with dexamethasone (100nM) and/or mifepristone (1μM) for 48 h. For AMPK activation experiments, confluent cells were treated for 1 h with serum-free growth medium containing 10mM 2-Deoxy-D-glucose (Sigma) or growth medium alone, before harvesting for Western blotting. For Wnt activation experiments, shControl and sh*HOTAIR* cells were differentiated in 96-well plates in the presence of CHIR99021 (1μM or 3μM, Abcam) or vehicle (DMSO). For measurement of intracellular lipids the cells were assayed using AdipoRed reagent (Lonza) on a FLUOstar Omega microplate reader (BMG Labtech). For insulin signalling experiments the insulin response (relative ratio of pAkt to tAkt) was examined under adipogenesis: the differentiation medium was changed to basal medium without insulin (Dulbecco’s modified Eagle’s medium/nutrient mixture F12 Ham’s supplemented with 2mM glutamine, 100 units/mL penicillin and 100 μg/mL streptomycin) on differentiation day 7 for 24h. Cells were treated with insulin (0nM, 25nM, 50nM, 100nM) for 10 min and harvested immediately.

#### Quantification of intracellular triglyceride content

Differentiated preadipocytes were harvested in triglyceride lysis buffer (1% IGEPAL CA-630; 150mM NaCl; 50mM Tris-HCl, pH 8.0). Cell lysates were sonicated then heated to 95°C for 30 min. Samples were cooled to room temperature, vortexed and centrifuged (12000 x *g* for 10 min at 4°C). Triglyceride concentration was quantified in the supernatant using an enzymatic assay (TAG assay, Instrumentation Laboratories). Quality controls and blanks were run in parallel. Triglyceride concentration was normalised to protein content (DC protein assay, Bio-Rad).

#### Generation of constitutive *HOTAIR* knockdown

For knockdown studies, *HOTAIR* shRNA pool (iv009919) and scrambled shRNA (LVP015-G) lentiviral particles were purchased from Applied Biological Materials. _im_ASAT/_im_GSAT cells were transduced for 20 h with lentiviral particles in growth medium containing 8 μg/mL hexadimethrine bromide (Sigma). Lentiviral medium was changed to normal growth medium for a further 24–48 h and viral transduced cells were subsequently selected in puromycin (1–2 μg/mL) containing growth medium.

#### Gene expression analysis

Total RNA was extracted from cells or AT using TRI-Reagent (Collins et al., 2010). cDNA synthesis and real-time qPCR were performed as previously described (Todorcevic et al., 2017). The ΔCT values of target genes were normalised to the ΔCt (geometric mean) of stably expressed reference transcripts (*PPIA, 18S, IPO8* or *PSMB6)* (Neville et al., 2011). TaqMan assays are listed in Table S4.

#### Western blot analysis

For Akt signalling cell lysates were prepared in ice-cold lysis buffer containing 50 mM Tris-HCl pH8.0, 150 mM NaCl, 1% IGEPAL CA-630, 10 mM sodium fluoride, 1 mM sodium orthovanadate and protease inhibitors (Complete EDTA-free, Roche). For EZH2 and AMPK phosphorylation studies, protein was extracted from cells using ice-cold cOmplete lysis M EDTA-free buffer with phosphatase inhibitors (Roche, Welwyn Garden City, UK). Proteins (20 μg) were resolved by Tris-glycine SDS-PAGE, transferred onto polyvinylidene fluoride membrane (Bio-Rad) and immunoblotted with primary antibodies: anti-phosphorylated Akt (Cell Signalling #4060), anti-total Akt (Cell Signalling #4685), anti-AMPKa (1:1000; Cell Signalling #5831), anti-pAMPK Thr172 (1:1000; Cell Signalling #2535), anti-EZH2 (1:1000; Cell Signalling #5246), anti-pEZH2 Thr311 (1:500; Cell Signalling #27888 ), anti-α-tubulin (1:5000; ab15246, Abcam) followed by horseradish peroxidise (HRP)-conjugated secondary antibodies: goat anti-rabbit IgG (Agilent Dako #P0448) or goat anti-rabbit IgG (H+L) (1:10,000; Invitrogen #31460). β-actin protein was probed with conjugated anti-actin (I-19) HRP (Santa Cruz Biotechnology, sc-1616 HRP). Detection was performed by enhanced chemiluminescence (Bio-Rad). Immunoblot images were captured on a Chemi-Doc XRS + (Bio-Rad) and analysed using ImageLab.

#### RNA immunoprecipitation (RIP)

RIP experiments were performed using EZ-Nuclear RIP (Cross-Linked) kit (Cat#17-10521, Millipore). Cells (1×10^6^/RIP) were cross-linked with 0.3% formaldehyde for 10 min. The cross-linked chromatin was incubated on ice and fragmented by frequent vortex for 30 min. Chromatin supernatant (10%) was collected as the input before adding the following immunoprecipitation antibodies: anti-EZH2 (Millipore #CS203195) and Mouse IgG (Millipore #CS200621). Magna ChIP Protein A/G Magnetic Beads (Cat#CS207374, Millipore) were used to bind the antibody/protein/RNA complex. RIP samples were washed, crosslinks were reversed and RIP RNA was purified. cDNA was synthesised for real-time qPCR using SYBR Green. For evaluating the interaction between lncRNAs (*HOTAIR, NEAT1)* and EZH2, the primers used were:

*HOTAIR* Forward: GGTAGAAAAAGCAACCACGAAGC,

*HOTAIR* Reverse: ACATAAACCTCTGTCTGTGAGTGCC,

*NEAT1* Forward: CTTCCTCCCTTTAACTTATCCATTCAC,

*NEAT1* Reverse: CTCTTCCTCCACCATTACCAACAATAC, and for the negative control *U1* snRNA primers were used:

*U1* Forward: GGGAGATACCATGATCACGAAGGT

*U1* Reverse: CCACAAATTATGCAGTCGAGTTTCCC

#### Chromatin immunoprecipitation (ChIP)

Cells (3×10^6^/ChIP) were cross-linked with 1% formaldehyde for 10 min. The cross-linked chromatin was sonicated (30sec ON/OFF for 10 min in a Bioruptor®Pico (Diagenode)) or enzymatically digested (EZ-Zyme^TM^ Chromatin Prep kit (Cat#17-375, Millipore) to generate <500 bp DNA fragments. Chromatin supernatant (1%) was collected as the input before adding the following immunoprecipitating antibodies: H3K4me3 (Diagenode C15410003), H3K27me3 (Diagenode C15410069 or Abcam ab6002), H3K27ac (Diagenode C15410174), H3K4me1 (Diagenode C15410037) and SUZ-12 (Abcam ab12073). Magnetic Dynabeads Protein A (Invitrogen) or protein G Agarose (EZ-ChIP^TM^ Chromatin Immunoprecipitation kit (Cat#17-371, Millipore)) were used to bind the antibody/antigen/DNA complex. ChIP samples were washed, crosslinks were reversed and ChIP DNA was isolated as the template for real-time qPCR using either SYBR Green (BioRad) or custom designed TaqMan assays (Tables S5 and S6).

#### RNA-seq analysis of sh*HOTAIR* cells

RNA samples were DNase treated using DNA-free DNA removal kit (Life Technologies) and RNA quality was analysed on a Bio-analyser 2100 (Agilent). The TruSeq paired-end RNA-seq libraries were prepared for a total of 18 samples (triplicates of sh*HOTAIR* and controls at D0, D4 and D14 during adipogenesis), and sequenced on Illumina HiSeq4000 at the Wellcome Trust Centre for Human Genetics, University of Oxford. The raw sequencing reads were mapped to the human genome hg19 using STAR version 2.5.1 (Dobin et al., 2013) with default settings. The GENCODE v19 GTF was applied to guide the spliced alignment. Duplicated alignments were marked with the MarkDuplicates script from the Picard tools v2.1.1 suite (http://broadinstitute.github.io/picard). Gene expression was quantified with featureCounts (Liao et al., 2014) using the GENCODE v19 GTF file. Differential expression analysis between the gluteal sh*HOTAIR* and shControl cells was performed at each time-point separately using DESeq2 (Love et al., 2014) with 5% FDR as the cut-off for reporting of the statistically significant results. Overlap between DEGs and curated gene sets was performed using CHEA (https://maayanlab.cloud/Harmonizome/resource/ChIP-X+Enrichment+Analysis) (Lachmann et al., 2010) and FUMA (https://fuma.ctglab.nl/) (Watanabe et al., 2017). Functional gene enrichment was analysed using the web-based software DAVID (http://david.abcc.ncifcrf.gov) (Sherman et al., 2022) and results presented with the term name and FDR-adjusted p*-*value. Gene expression patterns across adipogenesis (D0, D4, D14) were analysed using Weighted Gene Co-expression Network Analysis (WGCNA) (Langfelder and Horvath, 2008).

RNA-Seq data was used to assess differential alternative splicing (DAS) between sh*HOTAIR* and shControl cells. Trimmed reads were re-quantified at the transcript-isoform level with salmon (Patro et al., 2017) in pseudo-alignment mode, using the Human GENCODE GRCh37 (hg19) build as reference transcriptome. 3D-RNA-Seq (Guo et al., 2021) was used to explore isoform-level DAS (Table S3B). Data were first normalised using the Trimmed Means of M-Values (TMM) method following removal of transcript isoforms that did not have ≥1 counts per million reads (CPM) in ≥4 samples. DAS genes were those with at least one isoform changing in its percent splice-in (PSI) between sh*HOTAIR* and shControl by > 0.1 and (Benjamini-Hochberg Method) FDR <0.05. SUPPA2 (Trincado et al., 2018) was used to survey event-level DAS, specifying a --lower-bound change in PSI >0.1 (Table S3C). Lists of DAS genes were then uploaded to the gProfiler online g:GOSt tool (Raudvere et al., 2019) for overrepresentation analysis (Table S3D).

#### siRNA rescue experiments

Human siRNA oligo duplexes were used to transiently suppress *CEBPD* (Origene SR300761) or *PTEN* (Origene SR321496) from differentiation day4 (siRNA was added once on day4). Each kit contains three unique 27mer siRNA duplexes and one scrambled negative control siRNA duplex. The three siRNAs for each gene were tested and the one with best knockdown efficiency was chosen. siRNA and lipofectamine 2000 (Invitrogen) were mixed in antibiotic- and serum-free Dulbecco’s modified Eagle’s medium/nutrient mixture F-12 Ham (Sigma), then added dropwise onto cells cultured in antibiotic-free maintenance medium (differentiation or growth medium depending on the experiment type) for 20hr transfection. The scrambled siRNA was used as a control.

#### *HOTAIR* siRNA and TOPflash luciferase reporter assay

Four custom *HOTAIR* siRNA oligo duplexes, with target sequences matching the shRNA pool used to generate the *shHOTAIR* cells, were designed and pooled (Dharmacon). _im_GSAT cells expressing the 7TFP (TOPflash) lentiviral reporter vector (Fuerer and Nusse, 2010), as previously described (Loh et al., 2015a), were grown (5 x 10^4^) in 24-well plates for 24 h in antibiotic-free growth media then transfected with Lipofectamine RNAiMAX and either the pooled *HOTAIR* siRNA pool or Lincode Non-targeting negative control (Dharmacon) diluted in serum-free Optimem. Cells were cultured for 72 h prior to assessing Wnt signalling activity. The Luciferase Assay System (Promega) was used to measure TOPflash reporter activity on a Veritas Microplate Luminometer (Turner Biosystems). Luciferase results were corrected to cell protein measured with the Pierce Rapid Gold BCA Protein Assay (ThermoFisher).

#### Allele-specific qPCR to assess rs1443512 eQTL effect

Allele specific qPCR was performed essentially as described in Neville et al. (Neville et al., 2019). A dual-labelled TaqMan genotyping assay (Applied Biosystems) for SNP rs12312094 (C_2104255_20) was selected which is in LD with the body fat distribution associated SNP rs1443512 (Shungin et al., 2015) and falls within the *HOTAIR* transcript. Individuals heterozygous (n = 26) for the rs1443512-rs12312094 haplotype were identified from a cDNA panel of 204 GSAT samples using the PHASE v2.1.1 software (Li and Stephens, 2003) along with 6 individuals heterozygous for rs12312094 but not rs1443512. Genomic DNA (gDNA) for these individuals was also retrieved and diluted to 1.5ng/μL. The gDNA was used as the control comparison to the cDNA samples as there is an equal quantity of both alleles in heterozygous gDNA samples. By comparing the ratio of the Ct values from each allele (the ratio of the genotype assay Vic or Fam fluorophore signals) between cDNA and gDNA any allelic expression differences observed in the cDNA samples can be resolved. Any allelic imbalance between the two alleles in GSAT cDNA compared to genomic DNA from the same individuals was considered indicative of an eQTL effect. Data are presented as the percentage of the minor allele Ct value compared to the major allele Ct. This is calculated by first generating a standard curve and regression statistic for each assay. A standard curve is generated from genomic DNA for individuals homozygous for the major allele (BB) and minor allele (bb). Genomic DNAs are diluted to 1.5ng/μL then BB and bb homozygotes are combined to ratios 80:20, 60:40, 50:50, 40:60, 80:20. Following qPCR analysis using the dual-labelled TaqMan Genotyping assays the ratio of the B to b Ct values are calculated (Ct B minus Ct b) then plotted against the percentage of the minor allele in the dilution series. The linear regression statistic from this standard curve is then used to calculate the percentage minor allele expression of the unknown heterozygous individuals.

#### Genetic association analyses of rs1443512 with DXA-derived fat compartments

A meta-analysis of 25,276 individuals was performed to assess the association of rs1443512 with gynoid, leg, abdominal subcutaneous and abdominal visceral fat based on DXA-derived body composition. Details of the participants are provided in experimental model and subject details. Regional fat masses were natural log-transformed and adjusted for age, total fat mass and study specific covariates. The residuals after adjustment were rank-based inverse normally transformed for men and women separately. Genome-wide association studies were adjusted for the first 4 genetic principal components and conducted using BGENIE v1.2 (Bycroft et al., 2017) for Fenland and EPIC-Norfolk and using SNPTEST (Marchini et al., 2007) in the Oxford Biobank. For ORCADES, a genetic relatedness matrix based on identity-by-state inferred from the genotyping array was calculated and phenotypic residuals were calculated using GenABEL (Aulchenko et al., 2007). Genome-wide association analyses adjusted for genotyping array were run using RegScan (Haller et al., 2015). For UK Biobank, GWAS adjusted for the first 10 principal components and genotyping chip were run using BOLT-LMM (Loh et al., 2015b). Meta-analyses of the summary statistics describing the association of rs1443512 with fat depots were conducted using “metafor” in R (Viechtbauer, 2010).

### Quantification and statistical analysis

Evaluation of statistical significance was performed in SPSS 22.0, Graphpad Prism software and R scripts. All statistical details for experiments can be found in the figure legends and the results section.

## Data Availability

• sh*HOTAIR* RNA-seq data have been deposited at Gene Expression Omnibus (GEO) and are publically available as of the date of publication. The accession number is listed in the key resources table. Original western blot images have been deposited at Mendeley Data and are publicly available as of the date of publication. The DOI is listed in the key resources table. • This paper does not report original code. • Any additional information required to reanalyze the data reported in this paper is available from the lead contact upon request.

## Supplemental information

Document S1. Figures S1–S7 and Tables S4–S6

Table S1. Differentially expressed gene (DEG) lists (A) Number of differentially expressed genes (DEGs) in sh*HOTAIR* cells versus shControl. (B) Day 0; sh*HOTAIR* versus shControl. (C) Day 4; sh*HOTAIR* versus shControl. (D) Day 14, sh*HOTAIR* versus shControl.

Table S2. Functional gene enrichment analysis (A) Up-regulated day 0 gene overlaps between sh*HOTAIR* DEG list and gene sets from the Molecular Signature Database chemical and genetic perturbations (CGP). (B) Up-regulated day 0 gene overlaps between sh*HOTAIR* DEG list and EZH2 target genes extracted from ChIP-X enrichment analysis (CHEA). (C) Weighted gene correlation network analysis (WGCNA) modules. (D**)** Day 0 gene enrichment analysis (619 genes padj < 0.05; 578 DAVID annotated genes. Thresholds - counts:5 - EASE: 0.05). (E) Day 14 gene enrichment analysis (3,000 downregulated DAVID annotated genes padj < 0.05. Thresholds - counts:5 - EASE: 0.05). (F) Day 14 gene enrichment analysis (3,000 upregulated DAVID annotated genes padj < 0.05. Thresholds - counts:5 - EASE: 0.05).

Table S3. Alternative splicing (A) Event-level differential alternative splicing between shControl and sh*HOTAIR* cells identified by SUPPA2 (lower-bound change in PSI > 0.1). (B) Differential transcript usage statistical testing. (C) Differential alternative splicing gene-level statistical testing. (D) Overrepresentation analysis using gProfiler online g:GOSt.

Document S2. Article plus supplemental information
