# Supplementary material for: HOTAIR interacts with PRC2 complex regulating the regional preadipocyte transcriptome and human fat distribution
Source: Cell Rep. 2022 Jul 28;40(4):111136. doi: 10.1016/j.celrep.2022.111136 (PMC10073411; doi:10.1016/j.celrep.2022.111136)
Supplement: Document S1. Figures S1–S7 and Tables S4–S6 [file mmc1.pdf]

Cell Reports, Volume *40*

Supplemental information

HOTAIR interacts with PRC2 complex regulating the regional preadipocyte transcriptome and human fat distribution

Feng-Chih Kuo, Matt J. Neville, Rugivan Sabaratnam, Agata Wesolowska-Andersen, Daniel Phillips, Laura B.L. Wittemans, Andrea D. van Dam, Nellie Y. Loh, Marijana Todorčević, Nathan Denton, Katherine A. Kentistou, Peter K. Joshi, Constantinos Christodoulides, Claudia Langenberg, Philippe Collas, Fredrik Karpe, and Katherine E. Pinnick

Kuo_Supplementary_Figure_S1

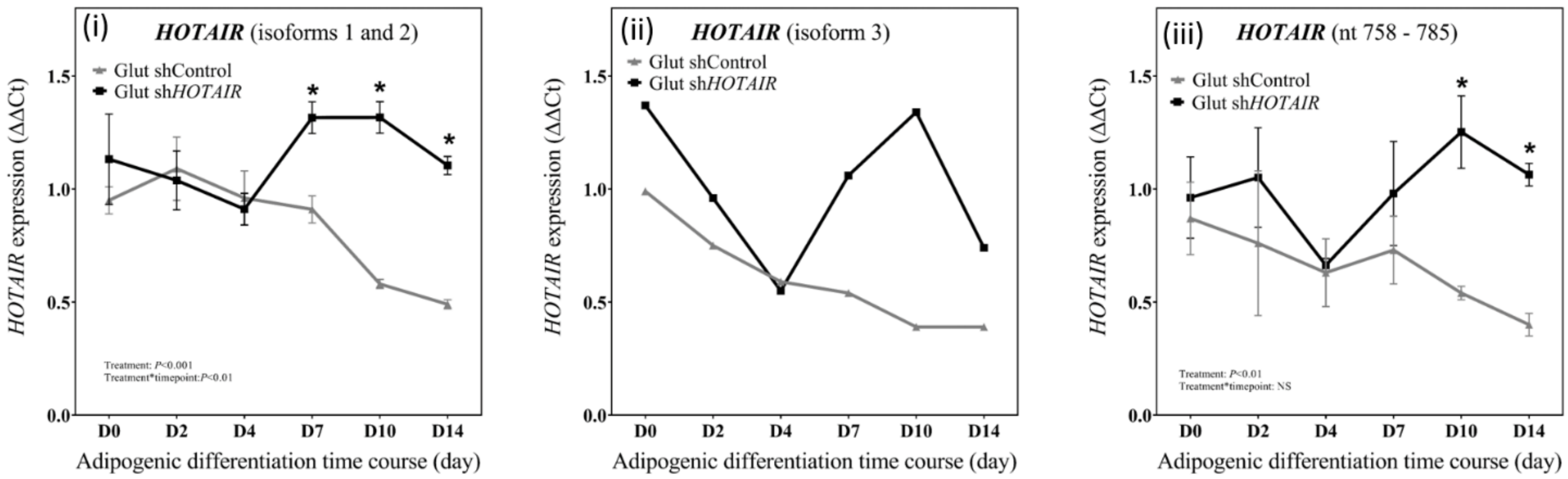

Figure S1: ***Assessment of HOTAIR expression in shHOTAIR cells,*** **Related to Figure 3A.** **(i)** *HOTAIR* expression (*HOTAIR1* and *HOTAIR2*; NR_047517 and NR_003716) in gluteal shControl (grey) and sh*HOTAIR* (black) preadipocytes during adipogenesis (n=3). **(ii)** *HOTAIR* expression (*HOTAIR3*; NR_047518) and **(iii)** amplification of *HOTAIR* region containing shRNA cleavage sites (nt 758 - 785). Statistical significance was assessed using two-way ANOVA, *$p<0.05$, shControl vs. sh*HOTAIR*

Kuo_Supplementary_Figure_S2

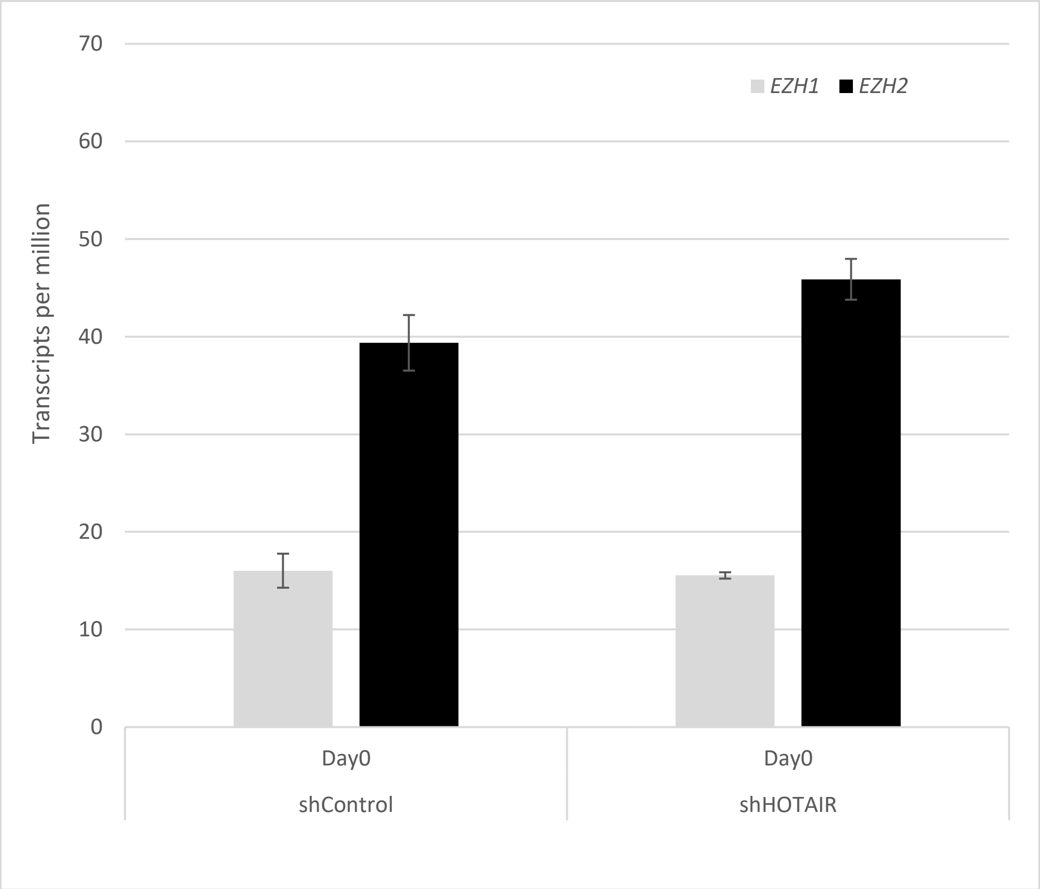

Figure S2: ***Expression of EZH1 and EZH2 in shControl and shHOTAIR preadipocytes*, Related to Figure 3B.** Data are expressed as transcripts per million (TPM) from RNA-seq analysis, mean ± SEM.

Kuo_Supplementary_Figure_S3

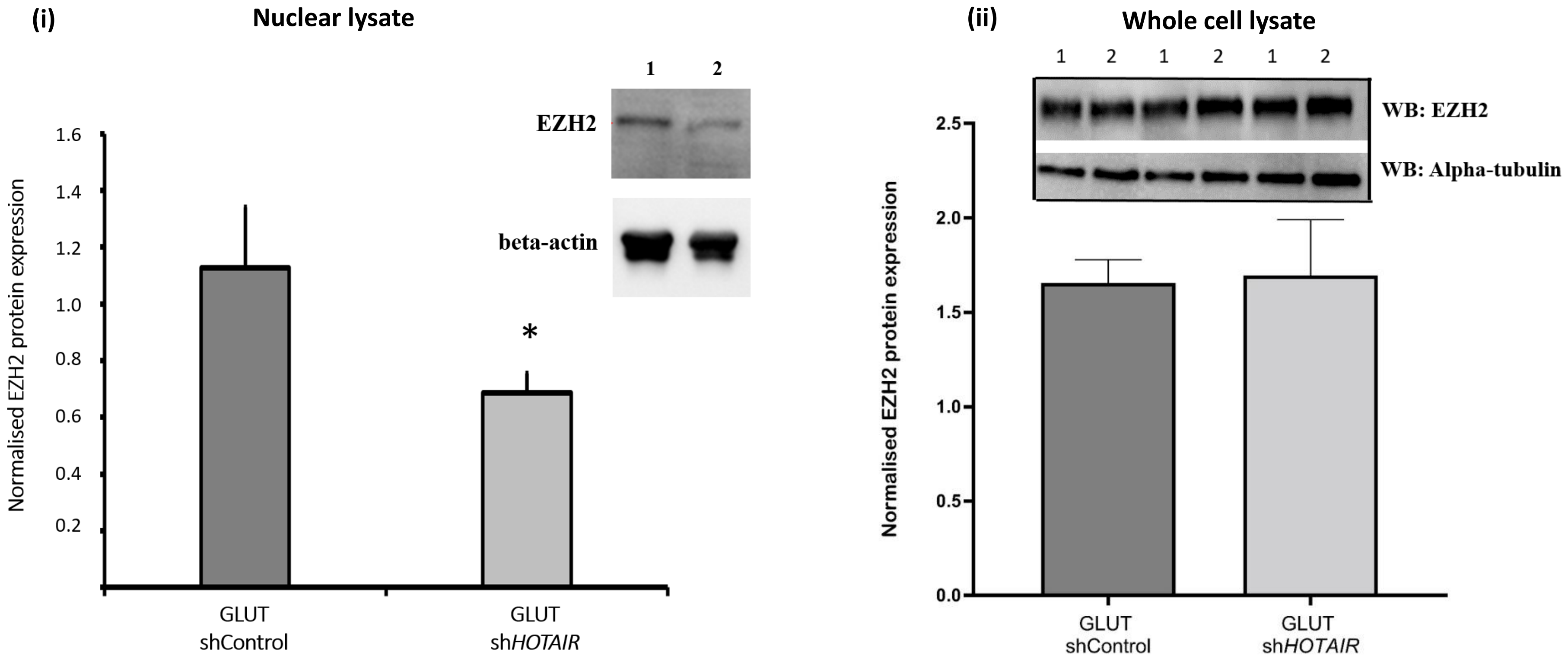

Figure S3: ***Expression of EZH2 protein in undifferentiated shControl and shHOTAIR cells,*** **Related to Figure 3B.** Representative Western blots and quantification showing detection of normalised EZH2 protein in (i) nuclear lysates used for subsequent RNA-IP analyses and (ii) whole cell lysates (lane 1: shControl, lane 2 sh*HOTAIR*). Statistical significance was determined by Wilcoxon signed ranks test (*p<0.05, n=3), mean ± SEM.

Kuo_Supplementary_Figure_S4

(i)

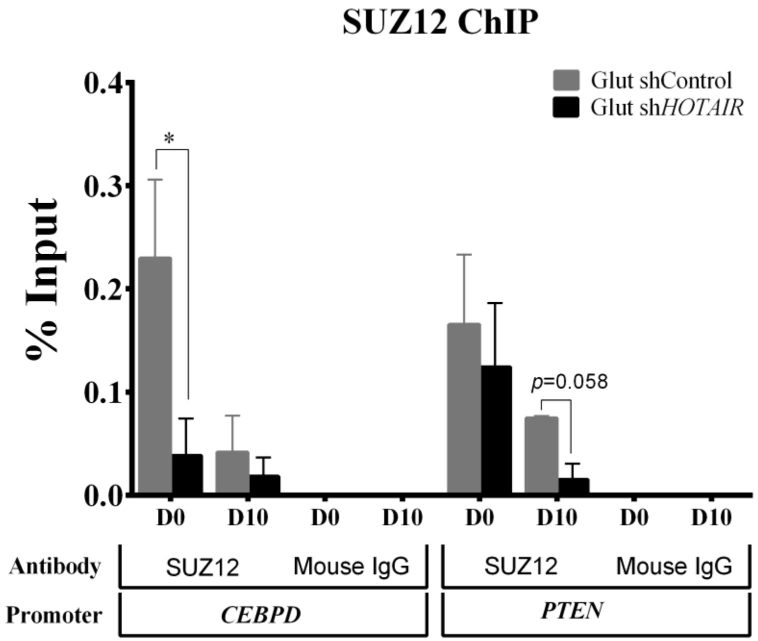

(ii)

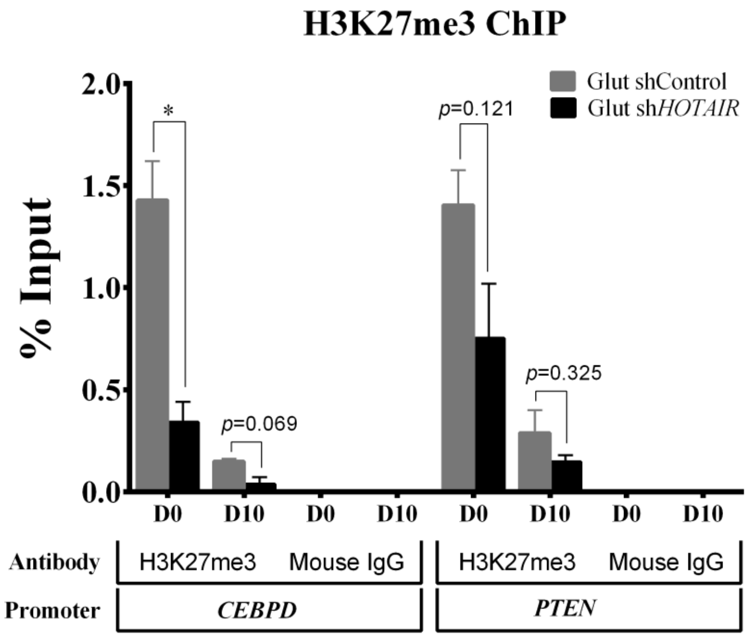

Figure S4: ***Regulatory modification elements found in CEBPD and PTEN promoters,*** **Related to Figure 4. (i)** Anti-SUZ12 and **(ii)** anti-H3K27me3 ChIP analysis in sh*HOTAIR* and shControl cells on differentiation day0 and day10 (n=3). Mouse IgG was used as a negative control. Data were assessed with independent or paired two-tailed T-test to compare levels between two cells, *$p<0.05$, mean ± SEM.

Kuo_Supplementary_Figure_S5

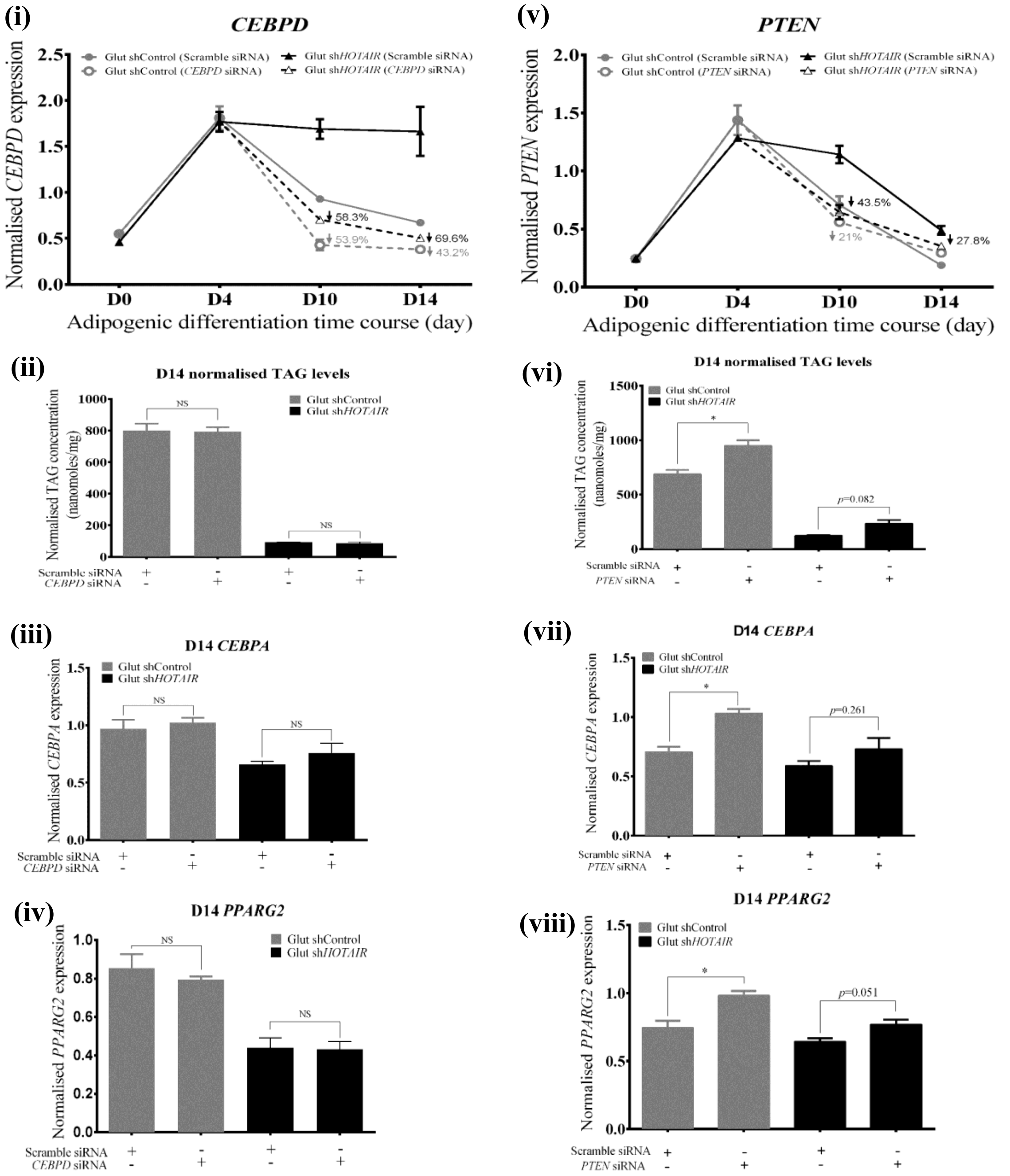

Figure S5: ***Recovery experiments after CEBPD or PTEN siRNA treatment of shHOTAIR cells,*** **Related to Figure 4. (i)** *CEBPD* expression in sh*HOTAIR* (triangles) and shControl (circles) cells treated with *CEBPD* siRNA (open) or Scramble siRNA (closed). **(ii)** Intracellular TAG content normalised to protein. **(iii)** *CEBPA* and **(iv)** *PPARG2* expression on differentiation day14 (n=3). **(v)** *PTEN* expression in sh*HOTAIR* (triangles) and shControl (circles) cells treated with *PTEN* siRNA (open) or Scramble siRNA (closed). **(vi)** Intracellular triglyceride TAG content normalised to protein. **(vii)** *CEBPA* and **(viii)** *PPARG2* expression on differentiation day14 (n=3). Data were assessed with independent two-tailed T-test, * $p < 0.05$, mean ± SEM.

Kuo_Supplementary_Figure_S6

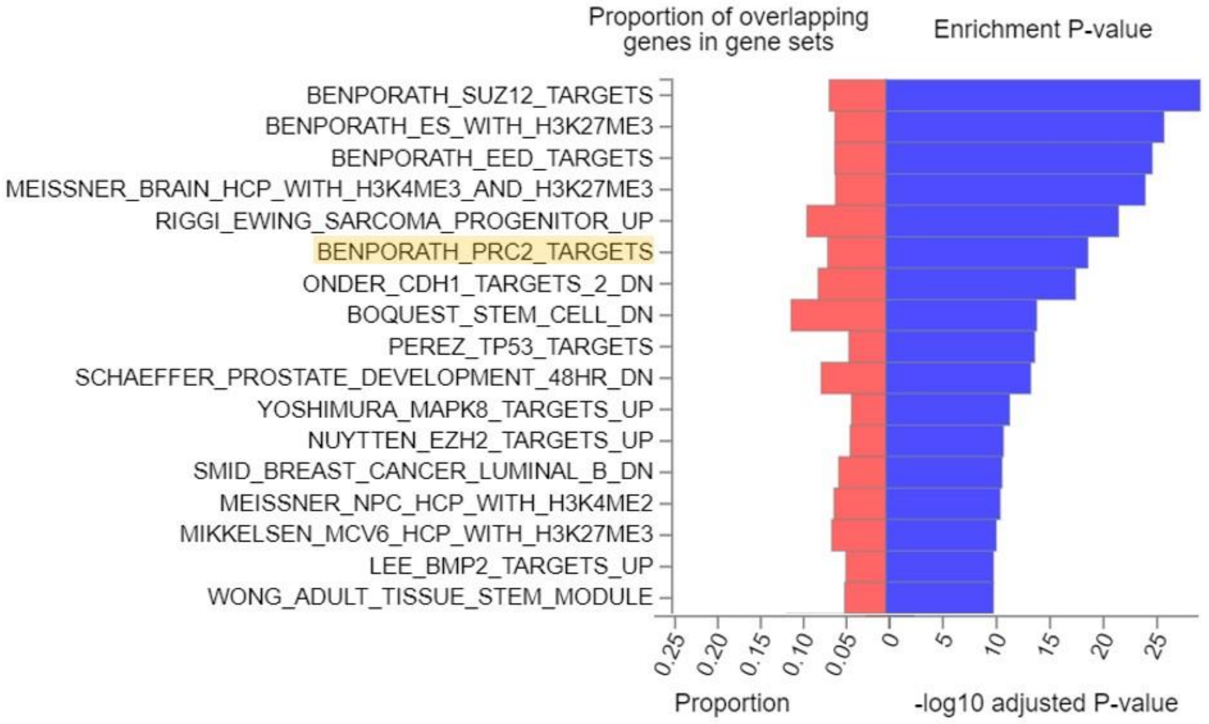

Figure S6: ***Overlapping genes between up-regulated Day0 shHOTAIR DEGs and gene sets from the Molecular Signature Database (CGP),*** **Related to Figure 5A.** Only gene sets where enrichment *p*-value was < -log10 are displayed.

Kuo_Supplementary_Figure_S7

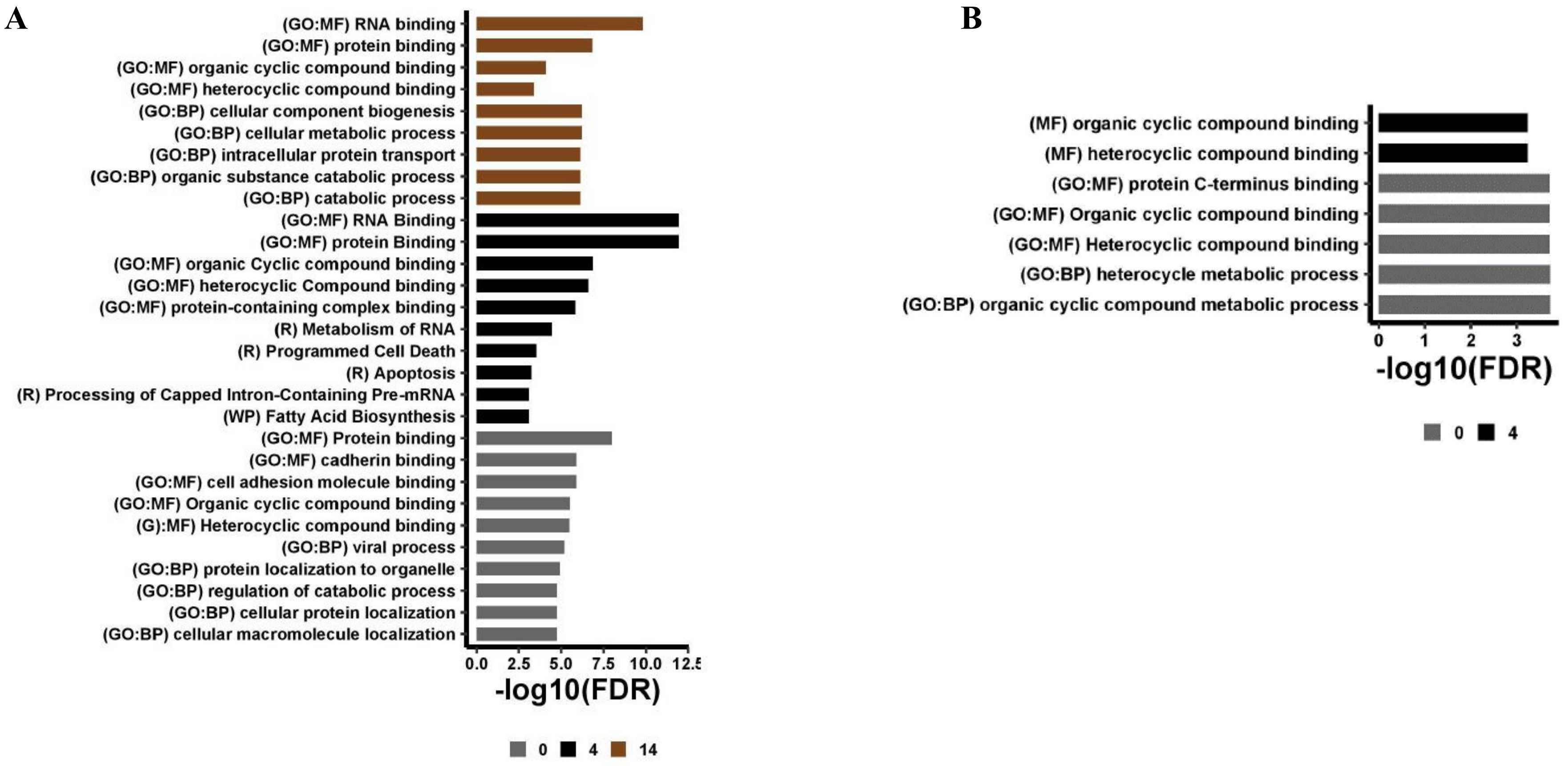

Figure S7. Functional enrichment of alternatively spliced transcripts, **Related to Figure 5.** Bar plots show significantly over-represented (FDR < 0.001) pathways and Gene Ontology (GO) terms for differential alternative splicing occurring at both the **(A)** isoform-level and **(B)** event-level in gluteal preadipocytes 0, 4, and 14 days after sh*HOTAIR*. Abbreviations: BP, biological process; MF, molecular function, CC, cellular component; R, Reactome pathways database; WP, Wikipathway pathways database.

Kuo_Supplementary_Table_S4

**Genes**	**TaqMan assay ID (Thermo Fisher Scientific)**
*HOTAIR**	Hs03296631_m1 *(HOTAIR1, HOTAIR2)*
	Hs03296680_s1 *(HOTAIR1, HOTAIR2, HOTAIR3)*
	Hs03296661_mH *(HOTAIR3)*
	Hs03296630_m1 *(HOTAIR1)*
	HOTAIR custom assay (nt 758-785):<br>Forward primer: ACAGAGAGAATAATCCGGGTCCTA<br>Probe: CGCCACATGAACGC<br>Reverse primer: CCCGCTCAGGTTTTTCCA
PPIA	Hs99999904_m1
18S	Hs99999901_s1
IPO8	Hs00183533_m1
PSMB6	Hs00382586_m1
CEBPA	Hs00269972_s1
CEBPB	Hs00270923_s1
CEBPD	Hs00270931_s1
PPARG2	Hs01115510_m1
HOXD8	Hs00251905_m1
HOXD10	Hs00157974_m1
PTEN	Hs02621230_s1
PCDH10	Hs00252974_s1
PAX3	Hs00240950_m1
HOXD3	Hs00232506_m1
HOXD9	Hs00610725_g1

Table S4: ***TaqMan assays used for gene expression,* Related to STAR Methods.**

Kuo_Supplementary_Table_S5

Amplicons	Forward primer	Reverse primer
R1	CACAAGTACACGCACCGGAA	GAGTGTTCACGAGTGGAGGC
R2	ACCCAGGCATCTGATCGCTA	TGATCAAAGTGAGCTCGCGG
R3	CTGCGAAGTGCTCCGAACTG	GGATGGAGGTTTTGGACCCG
R4	CTTTTTCCAAGCAGTGCCCC	CCTCCACCACCCCAATCTTC
R5	CAAGTGGCCTGATTGGAAG	GCGAATGTGGGTAGCTGAAT
R6	CCGCCCCCTTAGACCAAAAA	GGGGAGGAAAGTTAGGGGGA
R7	CAAAGCCCCGGAAGAATAGC	GGGCCTGCGCCTTATCATTT
R8	CTTTGTCCGTCCCCGAAAGA	CTCCAAATCTCGCTGTGGGT
R9	ACCAGTGGCAACTCTAAGCC	GGGCTGACAAAGCCCCTTAT
R10	ATGAAAGGAGGGCAGGAAC	CAAGAACTCGGTATTATGACATCAC
R11	GGAAACTGAGCTCTCGCCTG	CCGGCTGGAGCTGTAATGAA

Table S5: ***ChIP primers for exploring histone marks in the HOTAIR/HOXC locus,*** **Related to STAR Methods.** Eleven regions (designated R1-11) were selected for ChIP: R1-R2 are in the promoter region of two *HOTAIR* short transcript variants (NR_003716 and NR_047518), R3-R4 are in the promoter region of the *HOTAIR* long transcript variant (NR_047517) and R5-R11 are defined as *HOTAIR* putative enhancers in the FANTOM (Functional ANnoTation Of the Mammalian genome) database

Kuo_Supplementary_Table_S6

Custom TaqMan	Primers/Probe	Sequence
ChIP-*HOXD8*	Forward primer	CGGGCAGAGGGTGTTTTTT
	Reverse primer	CACTCTGGCCTCGGTTTACAA
	Probe	CCCTCCAGAGCCGG
ChIP-*HOXD10*	Forward primer	CACCCCCTGGTCTCTTTGC
	Reverse primer	TGGTATAGGGACAGCGCTTTTT
	Probe	TTGCAGTTGCCCCCCAG
ChIP-*PCDH10*	Forward primer	GCGAGCTCTCCAAAGCAAAA
	Reverse primer	TCTCCTAGCGGCGAAATCAG
	Probe	TCTCTGGCCTGCCC
ChIP-*CEBPD*	Forward primer	AGGAGCGAGGAGGTTCCAA
	Reverse primer	AGCACTCCAGGGCCTTCTC
	Probe	CCCACAAACAGGAAGA
ChIP-*PTEN*	Forward primer	TCCTACCGTTCCGTACTTTCCA
	Reverse primer	CCGGACCGTGCACGTT
	Probe	TCAACCCGGTAACCC

Table S6: ***Custom TaqMan assays designed for the promoter regions of genes,*** **Related to STAR Methods.**
