# Supplementary material for: HOTAIR interacts with PRC2 complex regulating the regional preadipocyte transcriptome and human fat distribution
Source: Cell Rep. 2022 Jul 28;40(4):111136. doi: 10.1016/j.celrep.2022.111136 (PMC10073411; doi:10.1016/j.celrep.2022.111136)
Supplement: Document S2. Article plus supplemental information [file mmc5.pdf]

Cell Reports, Volume *40*

Supplemental information

HOTAIR interacts with PRC2 complex regulating the regional preadipocyte transcriptome and human fat distribution

Feng-Chih Kuo, Matt J. Neville, Rugivan Sabaratnam, Agata Wesolowska-Andersen, Daniel Phillips, Laura B.L. Wittemans, Andrea D. van Dam, Nellie Y. Loh, Marijana Todorčević, Nathan Denton, Katherine A. Kentistou, Peter K. Joshi, Constantinos Christodoulides, Claudia Langenberg, Philippe Collas, Fredrik Karpe, and Katherine E. Pinnick
